# Supplementary material for: Single‐cell profiling screen identifies microtubule‐dependent reduction of variability in signaling
Source: Mol Syst Biol. 2018 Apr 4;14(4):e7390. doi: 10.15252/msb.20167390 (PMC5884679; doi:10.15252/msb.20167390)
Supplement: Supplementary file 1 — Appendix [file MSB-14-e7390-s001.docx]

**Appendix to: Single-cell profiling screen identifies microtubule-dependent reduction of variability in cell signaling**

Pesce, C. G^3^., Zdraljevic, S.^3^, Peria, W., Bush, A.^2^, Repetto, V.^2^, Rockwell, D., Yu, R. C.^3^, Colman-Lerner, A.^2^, and Brent, R.^1^

**Table of contents**

**1. Yeast strains** 2

**1.1 General construction methods** 2

**1.2 Strains**  3

**1.2.1 Construction of SGA88, the MATα partner to be mated with the nonessential haploid**

**deletion collection** 3

Track 1. Construction of the MATa parent of SGA88 3

Starting strain 3

Replacement of the BAR1 ORF with a CFP pheromone-responsive reporter

and a selectable marker 3

Replacement of the PRM1 ORF with a YFP pheromone-responsive reporter

and a selectable marker 4

Change of mating type to MATa 5

Change of ∆bar1 marker from URA3 to HIS3 5

Addition of ∆can1::P_MFA1_-LEU2 marker 6

Change in the DNA elements near the PPRM1-CFP reporter to correct for decreased

total expression and greater expression variability caused by colliding polymerases 7

Introduction of a constitutive control reporter 7

Track 2. Construction of the MATα parent of SGA88 9

Starting strain 10

Insertion of a selectable cdc28-as2 allele 10

Insertion of a P_PRM1_-mRFP reporter 11

Generation of SGA88 from Track 1 and 2 strains 11

**1.2.2 Construction of a gene deletion library containing required reporters and selectable markers** 12

Mating of SGA88 with the deletion collection, and subsequent sporulation 13

Diploid selection 13

Haploid selection 13

Selection of clonal haploids and assembly of the library for the flow cytometry-based screen 14

**1.2.3 Strains used for follow up studies** 15

Construction of GPY1802 15

Construction of GPY1804 15

Construction of GPY4000 16

Deletion of BIM1, GIM4 and other genes in GPY400, for follow up studies 16

Construction of SGA101, a reference and base strain for testing additional genes that

might affect microtubule function 17

Construction of SGA101 derivatives with genetic alterations in KAR1, KAR3, and CIK1 17

kar1-∆15 (SGA109) 17

kar3-1 (SGA108) 18

∆kar3 (GPY4003) 18

∆cik1 (GPY4123) 18

Construction of strains with nuclear label and gene expression reporters for nuclear

movement experiments 19

HTB2-YFP strains 19

SEC8-RFP SPC42-GFP strains 20

Construction of strains expressing human estrogen receptor chimeras 21

Vectors for homologous recombination with excisable CEN/ARS cassettes 21

Plasmid carrying estrogen receptor chimera driven by the ADH1 promoter 21

Plasmid carrying estrogen receptor chimera driven by the low variability BMH2 promoter 21

Strains carrying single copies of P_BMH2_-GEV or P_ADH1_-GEV 22

Comparison of variability of activation by P_BMH2_-GEV or P_ADH1_-GEV constructs 23

Construction of an estradiol inducible tub1-828-expressing strain (GPY1873) 24

Construction of strains expressing ectopic PRS activating proteins for bypass experiments 24

STE4 activator strains 25

STE5-CTM ∆ste5 strains 25

Construction of strains to quantify Ste5 membrane localization 26

**2 High throughput screen and follow up studies** 26

**2.1 Assembly of 96-format arrays of collection haploids isolated from single colonies** 26

Selection of genes included in the primary screen 26

Set 1: Unbiased Genes 27

Set 2: Kinases and Phosphatases 27

**2.2 High throughput growth, pheromone stimulation and flow cytometry measurements** 27

Growth to exponential phase 27

Induction of the pheromone response system 28

Flow cytometric measurements28

**2.3 Quantifying different contributions to cell-to-cell variability in cell signaling and response**  29

Analytic framework 30

Derivation of formula for estimation of signal variability 31

Computing the progressive spread distribution (PSD) and the median progressive spread (MPS) 34

Quantifying the stability of individual time course trajectories: the crookedness index I_C_ 34

**2.4 Selection of candidates and secondary screens**  35

Selection of candidates from primary screen 35

Secondary screens 36

Repeated assays on the same independently isolated segregants 36

Assays on three independently isolated segregants. 36

Microscope cytometry assays for cell-cell variability 37

Protocol for microscopy assay 38

**2.5 Follow-up studies of effect of gene deletions and other perturbations** 38

Dose-response assays 38

Consideration of morphology of Δbim1 and Δgim4 strains 39

Chemical disruption of microtubule polymerization 39

Analysis of correlations between nucleus to signaling site distance and pathway output 40

Effects of microtubule perturbations on the position of the nucleus relative to the signaling site 40

Analysis of correlation between nuclear position and pathway output 41

Cross stimulatory interactions and positive feedbacks at the signaling site 42

**3 Appendix Figure Legends**  43

**4 References** 45

##

## **1. Yeast strains**

*1.1 General strain construction methods*

We performed DNA manipulation, and yeast work (Ausubel et al., 1987-2017; Guthrie and Fink, 1991). Unless otherwise noted, we grew cells in synthetic dextrose complete (SDC) media consisting of Brent Supplemental Media (MP Biomedicals, Solon, OH), yeast nitrogen base without amino acids and ammonium sulfate (BD, Franklin Lakes, NJ), and 2% dextrose (Sigma-Aldrich, St Louis, MO).

### 1.2 Strains

We describe constructions of these key strains and plasmids below. We show the markers of strains used in experiments and other key strains in Appendix Figure S1.

#### 1.2.1 Construction of SGA88, the MATα partner to be mated with the deletion collection.

We generated a library of haploid *MATa* non-essential gene deletion mutants each containing the genetic elements used to carry out the variability screen. To do so, we first constructed SGA88, a *MATα *strain containing the same elements (*P_PRM1_-mRFP*, *P_PRM1_-CFP*, *P_ACT1-_YFP*, *cdc28-as2* and *∆bar1*), each marked with a different selectable marker. SGA88 also carried a “cassette” (*∆can1::P_MFA1_-LEU2*) carrying a *MATa*-specific marker (*P_MFA1_-LEU2*) that allowed positive selection of descendant haploid *MATa* cells, and two recessive drug resistant markers *(∆can1* and *∆lyp1*) that allowed counter selection of *CAN1/can1* or *LYP1/lyp1* diploids and parental *CAN1 LYP1* *MATa* cells (see more on the selection process below).

We constructed SGA88 in two separate, parallel tracks. Track 1 resulted in a *MATa* strain. Track 2 began with and resulted in a *MATα* strain. We introduced a subset of the desired modifications into strains from these tracks and then combined them into a single haploid strain isolated after mating and sporulation.

##### **Track 1. Construction of the MATa parent of SGA88**

######

###### Starting strain

The starting strain for Track 1 was *MATα* strain Y3656 (Tong et al., 2004) carrying a *∆can1::P_MFA1_-HIS3*-- *P_MFalpha1_-LEU2* cassettein the BY4742 (*MATα*) background. BY4742 is *his3∆1 leu2∆0 ura3∆0 lys2∆0.*

###### Replacement of the BAR1 ORF with a CFP pheromone-responsive reporter and a selectable marker

We constructed plasmid pBUPC by inserting a 200 bp cassette into the HindIII site of pRS406 (Sikorski and Hieter, 1989). This cassette was composed of 100 bp of *BAR1* locus sequence immediately upstream of the start ATG codon (*P_BAR1_*), 100 bp of sequence immediately downstream of the stop codon (*T_BAR1_*) and a linker with an Ase I site in between them. The relevant segment of pBUPC had the following layout:

Hind III *–🡨 T_BAR1_ (3’ – 5’)—Ase I site—P_BAR1_ (5’ – 3’) 🡪–* Hind III – Eco RI,

where arrows denote direction of function of the noted promoters and terminators.

We then inserted a *P_PRM1_-CFP-T_ADH1_* pheromone transcriptional reporter cassette in the marked EcoRI site of pBUPC, yielding plasmid pBUPC-PRM1-YFP. When cut with Ase I, pBUPC-PRM1-YFP yielded the following linear DNA molecule:

*🡨T_BAR1_ (3’ – 5’)—--URA3🡪--- P_PRM1_-CFP-T_ADH1_🡪-- 🡨P_BAR1_ (3’ – 5’)*, where

*URA3* denotes the entire *URA3* locus, with its promoter and terminators, as carried in pRS406 (Sikorski and Hieter, 1989).

We transformed y3656 with AseI-linearized pBUPC to obtain strain SGA10. We confirmed proper integration at the *BAR1* locus by PCR and tested that the strain was phenotypically *bar1* and had a pheromone-inducible CFP reporter.

###### Replacement of the PRM1 ORF with a YFP pheromone-responsive reporter and a selectable marker

To continue on this track, we then introduced a second, different colored, pheromone-inducible fluorescent protein reporter in SGA10. To do this we replaced the PRM1 ORF with the following DNA segment, obtained by amplifying the YFP- NAT resistance cassette from a plasmid from the *e collection* by PCR (Goldstein and McCusker, 1999; Longtine et al., 1998b), resulting in.

*PRM1-5’ (-50 to –1 from ATG)— YFP-T_ADH1_🡪---NAT^r^🡪-- PRM1-3’ (+1 to +50 from STOP*)

We transformed this PCR product into SGA10, selected for NAT resistance and confirmed integration by PCR. We also confirmed that the resulting strain had now both CFP and YFP pheromone inducible reporters. We named this strain SGA30.

###### Change of mating type to MATa

Our initial plan had been to introduce the reporters into a *MATα* strain that would be mated to the deletion collection. Later, we realized that we could check that the pheromone reporters in such a strain were inducible by mating pheromone using a factor, but, because a factor is not very soluble and hard to work with, to calibrate the performance of the reporters for any screen, we would need to verify their performance in a *MATa* strain. We therefore decided to calibrate the reporters carefully in such a *MATa* background before moving further. To do so, we changed the *MATα* reporter strain to *MATa*. Το do so, we mated the *MATα* SGA30 strain with a *MATa* strain from the BY4741 (*MATa his3∆1 leu2∆0 ura3∆0 met15∆0*) - derived deletion collection (we used the *∆ace2::Neo^R^*strain)*.* We did not name nor store the resulting diploid. We sporulated it and by plating in the appropriate multi-selection plates, obtaining a segregant with the following phenotype:

*MATa*

*∆can1::P_MFA1_-HIS3*-- *P_MFalpha1_-LEU2*

*∆bar1*:: T_BAR1_ (3’ – 5’)—--URA3🡪--- P_PRM1_-CFP-T_ADH1_🡪-- 🡨P_BAR1_ (3’ – 5’)

∆*prm1*:: (P_PRM1_🡪)YFP-T_ADH1_---NAT r

*LYS2*

*met15∆0*

We named the resulting strain SGA31.

###### Switch of ∆bar1 marker from URA3 to HIS3

When making SGA31, we discovered that haploid colonies from the sporulated diploid growing on NAT-containing uracil-histidine drop-out plates were comprised of both uracil auxotrophic and prototrophic segregants. We hypothesized, and then demonstrated, that *ura3* auxotrophic segregants were able to scavenge uracil derived from their *URA3* neighbors in the same colony. Since our plan of generating a modified deletion collection depended on our ability to stringently select only strains that carried the *∆bar1::P_PRM1_-CFP* reporter, we decided to replace the *URA3* marker for this reporter with a *HIS3* marker. We knew that *HIS3* allowed clean selections, since we had never found *his-* *MATa* segregants (segregants not expressing *P_MFA1_-HIS3*) mixed in with the *HIS3-*expressing *MATa* segregants.

*Addition of ∆can1::PMFA1-LEU2 marker*

To switch the marker in *∆bar1* we first needed to switch the *P_MFA1_-HIS3*-- *P_MFalpha1_-LEU2* to a different cassette that did not rely on *HIS3* expression to select *MATa* cells. To this end we obtained strain Y3996 from Amy Tong and Charlie Boone. Y3996 carried the following haploid-selection locus:

∆can1::P_MFA1_-LEU2.

This haploid selection locus, by contrast to Y3645’s, did not allow for selection of *MATα* segregants, but rather only *MATa* segregants. However, since selection of *MATα* segregants was not necessary for our approach, we switched to this selection cassette, freeing the *HIS3* locus to be used to mark *∆bar1.* We therefore mated SGA31 with strain Y3996 and used appropriate selection to identity a segregant, SGA33, with the following elements:

*MATa*

*∆can1::P_MFA1_-LEU2*

*∆bar1:: (T_BAR1_ 🡨 )(3’ – 5’)—--URA3🡪--- P_PRM1_-CFP-T_ADH1_🡪-- (🡨P_BAR1_) (3’ – 5’)*

*∆prm1:: (P_PRM1_🡪*)YFP-T_ADH1_---NAT^r^*

*LYS2*

*met15∆0*

Where the parenthesis around the series of symbols *P_PRM_*_1_, *T_BAR1_* and *P_BAR1_*indicates an element (promoter or terminator), endogenous to the locus.

To replace the *URA3* marker in *∆bar1* with a *HIS3* marker, we amplified the entire *HIS3* locus from pRS403 (Sikorski and Hieter, 1989) by PCR, using primers that yielded this PCR product flanked by 200 bp of pRS backbone sequences on each side. These same sequences flanked the *URA3* locus in the *URA3* marker inserted in *∆bar1*. We introduced this linear *HIS3* PCR product into SGA33 by transformation and selected for histidine prototrophy. All of the selected colonies had lost the *URA3* marker and had retained the pheromone-inducible *P_PRM_*_1_-*CFP* reporter. The resulting *∆bar1* locus had the layout below.

*∆bar1:: (T_BAR1_🡨) (3’ – 5’)—--HIS3🡪--- P_PRM1_-CFP-T_ADH1_🡪-- (🡨P_BAR1_) (3’ – 5’)*

We named this strain SGA37.

###### Change in the DNA elements near the P_PRM1_-CFP reporter to correct for decreased total expression and greater expression variability caused by colliding polymerases

With the *MATa* strain SGA37 in hand, we obtained high quality single cell level data of the expression of the CFP and YFP pheromone inducible promoters. We compared the results with those from TCY3096, a well-characterized reference W303 background strain containing the same reporters,

which carried the CFP reporter in a different locus, and the YFP reporter in the same locus as in SGA37 (Colman-Lerner et al., 2005). We found that the CFP reporter, but not the YFP reporter, had reduced level of expression in SGA37. In addition, gene expression noise (η^2^(γ)) (Colman-Lerner et al., 2005) for the *PRM1* promoter was higher in SGA37 than in TCY3096. We hypothesized that these two differences in SGA37 might be due to transcription starting at the intact *BAR1* promoter colliding with convergent transcription starting at the *PRM1* promoter. We demonstrated that this was the case by deleting 300 bp of upstream *BAR1* sequences (the pheromone response elements in P_BAR1_ reach up to -273 from the ATG) and showing that the behavior of the P_PRM1_-CFP reporter changed to now match the behavior of the reporter in the W303 control strain.

To delete the *BAR1* promoter we amplified the *URA3* locus from pRS406 flanked with sequences from the upstream of *BAR1* and the terminator in the CFP reporter,as schematized in (1):

(1) *BAR1(-350 to –300)— URA3🡪—-T_ADH1_ (last 50 bp)*

This PCR product was meant to recombine with the *∆bar1* locus in SGA37, schematized in (2):

(2) *∆bar1:: (T_BAR1_🡨) (3’ – 5’)—--HIS3🡪--- P_PRM1_-CFP-T_ADH1_🡪-- (🡨P_BAR1_) (3’ – 5’)*

The final, modified *∆bar1* locus would thus have the structure shown in (3):

(3) *∆bar1:: (T_BAR1_🡨) (3’ – 5’)—-HIS3🡪-- P_PRM1_-CFP-T_ADH1_🡪—🡨URA3-BAR1 (-300 – -350) (3’ – 5’)*

We transformed the PCR product depicted in (1) into SGA37 and confirmed proper recombination by PCR. We named the resulting strain SGA39. As stated above, expression level and gene expression noise for the P_PRM1_-CFP-T_ADH1_ were restored to normal behavior by this procedure.

In a subsequent step, we removed the *URA3* marker by transforming SGA39 with a double stranded oligonucleotide with homology to the *BAR1* (-300 – -350) segment and the *URA3* gene terminator, and plating on 5-FOA containing plates. The resulting *∆bar1* locus kept the URA3 terminator as a safeguard against any possibly colliding polymerases that started transcription to the right of this construct. The layout of this locus was:

*∆bar1:: (T_BAR1_🡨) (3’ – 5’)—-HIS3🡪-- P_PRM1_-CFP-T_ADH1_🡪— 🡨T_URA3_ BAR1 (-300 – -350) (3’ – 5’)*

Where, as before, arrows, including 🡨 for the URA3 terminator, denote the functional orientation of the inserted sequences. There is no known transcription starting in the terminator sequences.

We named the resulting *∆bar1-ORF::PRM1pr-CFP--HIS3 ∆bar1-promoter::URA3-terminator* strain SGA41. SGA41 does not show the reduced expression and increased noise of the *P_PRM1_-CFP* reporter observed in parental strain SGA37. Rather, both *P_PRM_*_1_ reporters in SGA41 showed results identical to the same *P_PRM1_* reporters in the reference W303 strain TCY3096.

###### Introduction of a constitutive control reporter

At this point in the strain construction process, research in W303 strains had uncovered that cell-to-cell variability in the single-cell levels of expression from any transcriptional fluorescent protein reporter was substantially increased by a large cell-to-cell variability in the capacity of cells to express genes into proteins (Colman-Lerner et al., 2005). In addition, the same work had developed an internal-reference method using an additional different-colored fluorescent protein transcriptional reporter driven by the promoter of a housekeeping gene. This method allowed the separate quantification of η^2^(P), cell-to-cell differences in the strength of the signal received by the promoter (pathway variability). To allow these measurements, we decided to alter our plans for the Track 1 strain so that it included a third fluorescent protein reporter, driven by the promoter for the housekeeping gene *ACT1*.

At the same time, in parallel to these developments in our lab, the Boone lab had added a major improvement to the SGA pipeline by developing *∆lyp1* as a second selectable marker to eliminate unsporulated diploids in the haploid selection step (Tong et al., 2004). *LYP1* codes for a lysine transporter that is only required in lysine auxotrophs. This transporter (the Lyp1 permease) is the only means by which the toxic lysine analog thialysine can enter the cell, therefore the *∆lyp1* mutation is a recessive thialysine resistance mutation. Addition of both canavanine and thialysine to the haploid selection plates provides a “double lock” mechanism to prevent the growth of unsporulated diploids (after mating with the knock-out collection, all diploids have to be *CAN1/can1 LYP1/lyp1*).

We decided to combine our two new needs: for a *∆lyp1* mutation and for a constitutive fluorescent protein reporter by inserting the constitutive *P_ACT1_* reporter in lieu of the *LYP1* ORF.

Similar to what we had done above for *BAR1* and P_PRM1_-CFP, we therefore constructed pLYP1a, containing a 200 bp cassette in the HindIII site of pRS406. This cassette was composed of 100 bp of *LYP1* locus sequence immediately upstream of the start ATG codon (P_LYP_*_1_*), 100 bp of sequence immediately downstream of the stop codon (T_LYP1_) and a linker with an AflII site in between them. We then cloned a *P_ACT1_-YFP* cassette adjacent to the *LYP1* integration cassette, yielding the plasmid pLYP1a-PACT1-YFP. The scheme below represents pLYP1a-PACT1-YFP linearized with AflII

*(5’ – 3’)(P_LYP1_ 🡪)--URA3🡪--- P_ACT1_-YFP-T_ADH1_🡪-- (🡪T_LYP1_)(5’ – 3’)*

Note that in contrast to the pBUPC construct used for *BAR1* and *P_PRM1_-CFP*, in the *LYP1* *P_ACT1_-YFP* construct the coding strands are the same for *LYP1*, *URA3* and *ACT1* sequences, preventing the “collision” of polymerases that affected the *P_PRM1_-CFP* reporter (see above).

We transformed AflII-linearized p*LYP1a-PACT1-YFP* into SGA41 and selected for uracil prototrophy, yielding SGA43. We confirmed the correct insertion by PCR, by the acquisition of resistance to thialysine and by the fact that the cells constitutively expressed YFP.

##### **Track 2. Construction of the MATα parent of SGA88**

###### Starting strain

The starting strain for Track 2 was the *MATα* strain BY4742. As described below, we introduced into this strain a *cdc28-as2* allele and a *P_PRM1_-mRFP* reporter. We later combined these two components of the track 2 strain with components described above present in SGA43 by mating and meiotic segregation.

###### Insertion of a selectable cdc28-as2 allele.

We replaced the *CDC28* gene with the ATP analog (1-NM-PP1)-sensitive *cdc28-as2* (Bishop et al., 2000) allele using plasmid pCDC28-as2-406 (Colman-Lerner et al., 2005). Linearized pCDC28-as2-406 recombines with the CDC28 gene and integrates by a single recombination step.

The modified *CDC28* locus is schematized below:

(*P_CDC2_*_8_*🡪*)*cdc28-as2 ORF–T_CDC28_ --URA3🡪--(no ATG) CDC28 ORF-(🡪T_CDC28_)*

Note that the “(no ATG) *CDC28* ORF” not only lacks a start codon, it also lacks an upstream promoter.

We transformed linearized pCDC28-as2-406 into BY4742 and selected for uracil prototrophy. The resulting strain SGA60 was sensitive to the ATP analog 1-NM-PP1.

In our previous use of this construct we followed this transformation by selecting for “looped out” variants using 5-FOA selection, leaving an unmarked *P_CDC2_*_8_*🡪cdc28-as2* locus. For the purpose of this genetic screen we needed to have a selectable marker next to *cdc28-as2*, a marker different than the ones used before (*URA3* was occupied by the *∆lyp1* P_ACT1_-YFP element). We also needed to use a *cdc28-as2* element unable to revert to wild type *CDC28* by loop-out recombination.

We therefore replaced the URA3 marker and the WT *CDC28* ORF and terminator homology at the 3’ end of the modified locus using a PCR approach with a hygromycin B resistance cassette schematized here: *(🡪T_CDC28_) (+300 – +350)---HygB^R^--- (🡪T_CDC28_ +400 – +450)*

After a double recombination event with the PCR product, the structure of the *CDC28* locus would be as schematized below:

*(P_CDC28_🡪) cdc28-as2 ORF–T_CDC28_ –HygB^R^🡪--intergenic sequence,*

where the PCR product sequences are double underlined.

We transformed the *HygB^R^* PCR product into SGA60 and selected for hygromycin resistance to obtain strain SGA62. We confirmed proper insertion by PCR and by loss of uracil prototrophy. As expected, the resulting strain, SGA62 was sensitive to inhibition by the mutant kinase inhibitor 1-NM-PP1.

###### Insertion of a P_PRM1_-mRFP reporter

We constructed the *P_PRM1_-mRFP* reporter via the same PCR approach used above for the *P_PRM1_-YFP* reporter, replacing the *PRM1* ORF with the *mRFP* ORF followed by a selectable marker. We obtained an mRFP variant of the Pringle collection plasmids carrying the nourseothricin resistance (*NAT^R^*) selectable marker from the O’Shea lab (UCSF, (Huh et al., 2003)).

After transformation the modified *PRM1* locus has the structure schematized below:

*∆prm1:: (P_PRM1_🡪)mRFP-T_ADH1_---NAT^R^*

We transformed the *mRFP – NAT^R^* PCR product targeting *PRM1* into SGA62 and selected for NAT resistance. We confirmed integration by PCR and by the presence of a pheromone inducible mRFP. The resulting strain was named SGA64.

##### Generation of SGA88 from Track 1 and 2 strains.

We mated strains SGA43 and SGA64, yielding diploid DSGA1. We sporulated DSGA1, dispersed the spores and plated them in arginine and histidine dropout plates with canavanine, NAT and HygB. We replica-plated the resulting colonies onto different single-dropout plates that respectively lacked leucine, uracil, lysine and methionine.

We screened the uracil and methionine prototrophs and lysine and leucine auxotrophs (leucine auxotrophy identifies *MATα* cells) to find segregants with a pheromone-inducible RFP reporter (since half would have it, the other half would have the YFP variant).

We named the resulting strain SGA88. It carried the following genetic elements:

*MATalpha*

*∆can1::P_MFA1_-LEU2*

*∆bar1:: (🡪T_BAR1_) (3’ – 5’)—-HIS3🡪-- P_PRM1_-CFP-T_ADH1_🡪—URA3🡪- 🡨T_ura3_ BAR1 (-300 – -350) (3’ – 5’)*

*∆prm1:: (P_PRM1_🡪)mRFP-T_ADH1_---NAT^R^*

*P_LYP1_ (5’ – 3’)🡪--URA3🡪--- P_ACT1_-YFP-T_ADH1_🡪-- (🡪T_LYP1_) (5’ – 3’)*

*(P_CDC28_🡪*) *cdc28-as2 ORF–T_CDC28_ –HygB^R^🡪--*intergenic sequence

*LYS2*

*MET15*

We tested for the presence of each of these elements in SGA88 by PCR, and also using phenotypic assays when those existed.

We used SGA88 as the *MATα* partner to mate with the non-essential haploid deletion collection.

As a control *MATa* strain, we also selected a sibling of SGA88 that grew without leucine (and thus was *MATa*) and that had a pheromone-inducible mRFP reporter. This strain was *met15∆0* and was named SGA85.

We used SGA85, seeded in multiple wells on each 96 well plate, as the control strain in the high throughput variability screen, and as a reference strain for follow up studies.

#### 1.2.2 Construction of a gene deletion library containing required reporters and selectable markers

We constructed a ~4,000 strain gene deletion library in which each strain contained a deletion of a nonessential gene as well as the genetic elements described in Appendix Figure S1. To build this, we developed a modification of the procedure described by Charlie Boone and collaborators (Tong and Boone, 2007; Tong et al., 2004; Zhang et al., 2014). Appendix Figure S2 shows a cartoon of these steps.

##### Mating of SGA88 with the deletion collection, and subsequent sporulation.

Briefly, we first grew a lawn of the *MATα* partner SGA88 on rectangular YPD agar plates, pinned a freshly grown 384-format array of the yeast deletion collection on top of the lawn and incubated the plates for 24 h at 30^o^ C. We next pinned the grown, partially diploid colonies onto diploid selection plates and incubated for 2 days at 30^o^ C; this step eliminated the unmated haploids. We subsequently pinned the diploids onto sporulation plates and incubated for 5-8 days at 25^o^C. We monitored the progress of sporulation each day by counting the fraction of spore-containing asci in the colonies in the microscope. Once the number of asci had plateaued we stored the sporulated colonies at 4ºC until the next step.

The differences with the Tong and Boone (2004) procedure are described below:

###### Diploid selection.

We used antibiotic selection in G418 and HygB plates instead of relying on the auxotrophic markers *lys2∆0* and *met15∆0*.

###### Haploid selection.

To isolate single haploid deletion strains carrying the above reporters, we sporulated the diploid colonies and manually streaked from these onto haploid selection plates instead of pinning them as a patch on the selection agar (see next section). We did this because our screening method was aimed at detecting changes in standard deviation in single cell distributions, and thus we needed a very stringent approach to collect the strains that made up our library.

We used haploid selection medium that allowed us to positively select for a larger number of genetic elements than the process described by Tong and Boone (Tong and Boone, 2007). The counter selection of diploids in this medium was effected by canavanine (to kill all *∆can1*/*CAN1* diploids) and thialysine (to kill all *∆lyp1*/*LYP1* diploids).

##### Selection of clonal haploids and assembly of the library for the flow cytometry-based screen.

Appendix Figure S1 shows the genetic markers haploid strains needed to have to grow on these plates.

To maximize the chances that all the cultures in our screen would be genetically homogeneous, we manually picked colonies from these streaked plates. In several cases we found two or more populations of colonies on haploid selection plates distinguished by their size. We interpreted the size heterogeneity as a sign that the strain in the original haploid deletion library carried one or more genetic changes that relieved a growth defect caused by the gene deletion (for example aneuploidy (Hughes et al., 2000), second site suppressors (Hittinger and Carroll, 2007), or formation of same sex diploids (Giaever and Nislow, 2014)). To minimize the presence of such mutations in our manually isolated clones, we avoided the larger colonies and picked only smaller ones.

We inoculated each selected colony into liquid medium that lacked histidine and contained canavanine, thialysine, nourseothricin, and hygromycin (the same selection agents as the haploid selection plates) . We used 500 µl of media in 1.1 ml capacity “deep well” polypropylene 96-format plates. In each plate we also included the following controls:

SGA85: 4-6 wells per plate

BY4741: 4 wells per plate.

We distributed the placement of the control cells on the plates used for the screen in random patterns.

We then grew the 96-well plates to carbon exhaustion, 2 days at 30^o^C. We stored half of the saturated cultures at 4^o^C until the next step. We added 15% glycerol to the other half and preserved at -80^o^C.

#### 1.2.3 Strains used for follow up studies

*Construction of GPY 4000, the reference and base strain for follow up studies*

Construction of GPY1802

We first generated GPY1802, a BY4741 derivative with markerless *∆bar1* and *cdc28-as2* loci. To do so, we first deleted *BAR1* in BY4741 using a PCR product containing the pRS406 URA3 gene flanked by 50 nt of homology to the *BAR1* promoter and terminator. We confirmed proper insertion by PCR and a phenotypic test of *BAR1* status. We named the resulting *∆bar1::URA3* strain GPY8190.

We then deleted the *URA3* marker by transforming GPY8190 with a 100-bp double strand oligonucleotide composed of two “arms” of 50 nt each with homology to the sequences flanking the *URA3* marker. We selected for *∆ura3* transformants on 5-FOA plates and confirmed proper deletion by PCR. We named the resulting strain GPY1800 and annotated this locus in the strain table as *∆bar1::∆marker* (Appendix Table S1).

Next, we introduced the *cdc28-as2* allele. We used linearized pCDC28-as2-406 to “loop in” the allele, generating strain GPY1801, and then we “looped it out” as described earlier (Colman-Lerner et al., 2005). We confirmed proper recombination by PCR and confirmed it by testing for 1-NM-PP1 sensitivity. We named the resulting strain GPY1802 and annotated this marker in the strain table as *cdc28-as2* (Appendix Table S1).

Construction of GPY1804

We used GPY1802 to generate the *TUB1-828-expressing* strain and its cognate reference strain (see below). To do so, we added a *P_PRM1_-mCherry* reporter to GPY1802 by replacing the PRM1 ORF with the mCherry coding sequence followed by a terminator and a marker. To do so, we used an approach identical to the one above for *P_PRM1_-YFP* and -*mRFP*, except that in this case we used an *mCherry-T_ADH1_---G418^r^* template described before (Yu et al., 2008). We confirmed integration by PCR and checked the presence of a pheromone-inducible mCherry reporter. We named the resulting strain GPY1804, and annotated this locus as *PRM1pr-mCherry--G418(MX6)* in the strain table (Appendix Table S1).

Construction of base strain GPY4000

In other experiments, we had learned that *P_ACT1_*was mildly induced by pheromone. We therefore chose to use *P_BMH2_-YFP* as a constitutive promoter in the base strain for the follow up experiments. This ensured that the changes in η^2^(P) observed in the deletion mutants did not arise from a change in variability in the pathway controlling the constitutive control reporter.

To construct GPY4000, the reference strain for follow up studies, we first generated a plasmid carrying the reporter construct by modifying plasmid pTC-*P_BMH2_-YFP- T_ADH1_-URA3* (Colman-Lerner et al., 2005): we removed its *URA3* marker and replaced it with a genomic PCR product of the *MET15* gene, generating plasmid pTC-*P_BMH2_-YFP--T_ADH1_-MET15*. We linearized pTC-P_BMH2_-YFP-T_ADH1_-MET15 with StuI, which cuts within the *P_BMH2_* element, and transformed the linearized plasmid into GPY1804. We screened 8 methionine prototrophic (*MET*^+^) transformants using fluorescence microscopy to identify an integrant with a single copy of the *P_BMH2_-YFP* reporter. We named this strain GPY4000 and annotated this locus in the strain table as *BMH2::P_BMH2_-YFP--MET15* (Appendix Table S1).

##### Deletion of BIM1, GIM4, FUS3, KSS1 and other genes in GPY4000 for follow up studies

We introduced all gene deletions in the GPY4000 strain by a PCR approach. We first obtained strains carrying the gene deletion of interest marked with G418 resistance from the haploid deletion collection. We then changed the G418 resistance to either NAT resistance or hygromycin B resistance by transforming the deletion strain with a PCR product of the resistance cassettes and selecting for the marker carried in the PCR product. Since all three drug resistance markers contained the same 300-400 bp promoter and terminator, those flanking sequences directed a double homologous recombination gene replacement. Proper recombination was confirmed by the loss of the original G418 resistance. Subsequently, we amplified the gene deletion locus by PCR, including 300-500 bp of 5’ and 3’ gene-specific flanking sequences in the amplicon. We transformed this PCR product into GPY4000, selected for the appropriate drug resistance and confirmed proper gene deletion by PCR. To make double deletion strains, we repeated the process above, starting with a strain with one deletion. We transformed the one-deletion strain with a PCR product targeting a second gene and carrying a different selectable marker. We confirmed proper gene deletion by PCR. The resulting strains are listed in Appendix Table S1.

##### Construction of SGA103, a reference and base strain for testing additional genes that might affect microtubule function

SGA85 is the haploid *MATa* reporter strain used as a reference in these screens. For some follow up microscopy experiments requiring three color FP live cell time courses (see below), we used a derivative of it SGA101, as a base strain to introduce different alleles possibly affecting microtubule function by loop in/ loop out replacement with *URA3*-marked plasmids. Starting with the reference strain SGA85, we derived a *∆ura3* strain by transforming SGA85 with a PCR product spanning the entire *LYP1* locus from promoter to terminator, amplified from BY4741 genomic DNA. We selected for loss of the *URA3* marker on 5-FOA plates and confirmed restoration of the *LYP1* ORF by verifying the loss of YFP fluorescence from *P_ACT1_-YFP-T_ADH1_*and by diagnostic PCR. The resulting strain was a *ura3 LYP1* derivative of SGA85. We named it SGA101.

##### Construction of SGA101 derivatives with genetic alterations in KAR1, KAR3, and CIK1

We first constructed a wild type reference strain for this set of mutants. We transformed SGA101 with pTC-*P_BMH2_-YFP- T_ADH1_-URA3* linearized with StuI and identified transformants with a single *P_BMH2_-YF*P copy using fluorescence microscopy. The resulting strain was named SGA103.

kar1-∆15 (SGA109)

We obtained plasmid pMR1593 as a gift from Mark Rose and used it as described (Vallen et al., 1992) to replace *KAR1* for *kar1-∆15* in parent strain SGA101. Briefly, we linearized pMR1593 with BglII, transformed it into SGA101, selected for uracil prototrophy and in a second step selected for loss of the *URA3* marker on 5-FOA plates. We screened the resulting colonies by PCR to identify a *kar1-∆15* strain, which was named SGA107.

We then transformed SGA107 with pTC-*P_BMH2_-YFP- T_ADH1_-URA3* linearized with StuI as above. We identified transformants with a single *P_BMH2_-YFP* copy using fluorescence microscopy. The resulting strain was named SGA109.

kar3-1 (SGA108)

We obtained the plasmid pMR1510 from Mark Rose and used it as described to replace *KAR3* with *kar3-1*. Briefly, we linearized pMR1510 with MluI, transformed it into SGA101, selected for uracil prototrophy and in a second step selected for loss of the *URA3* marker on 5-FOA plates. We screened the resulting colonies by PCR to identify a *kar3-1* strain, which was named SGA106. As above, we transformed SGA107 with linearized pTC-*P_BMH2_-YFP- T_ADH1_-URA3* and identified transformants with a single *P_BMH2_-YF*P copy using fluorescence microscopy. The resulting strain was named SGA108.

∆kar3 (GPY4003)

We deleted *KAR3* in the SGA103 background, by PCR using pAG32, which has a hygromycin B resistance hph cassette. This PCR segment was transformed into SGA103, which was then plated on non-selective medium and replica plated to media containing hygromycin B. Transformants were confirmed to be knockouts of *KAR3* by PCR, and the resulting strain was named GPY1034.

∆cik1 (GPY4123)

We made this *∆cik1* strain from GPY4104, a base strain carrying the same fluorescent reporters and modifications as GPY4000, with the exception that the selectable marker next to *P_PRM1_-mCherry* was *URA3MX4* (Goldstein et al., 1999 1999) instead of *G418^R^*. To do this marker exchange we transformed GPY4000 with a PCR product of the *URA3MX4* cassette and screened the URA^+^ clones to find one in which the *URA3* ORF had replaced the *KAN^R^* ORF by homologous recombination among the promoter and terminator sequences shared by the two selectable markers.

*T*o make strains lacking the *CIK1* coding sequence, we PCR amplified the *KAN^R^(MX4)* cassette to generate a PCR product that had the *P_TEF_-G418^R^-T_TEF_* cassette with homologous tails directly flanking the 5’ and 3’ of the CIK1 ORF. GPY4104 was then transformed with the generated PCR product, plated on non- selective media, and replica plated to media containing geneticin. Transformants were confirmed to be knockouts of CIK1 by PCR and the resulting strain was named GPY4123.

##### Construction of strains with nuclear label and gene expression reporters for nuclear movement experiments

HTB2-YFP strains

In SGA85 and its derivative SGA101, the *P_PRM1_-CFP* reporter had been inserted in the *∆bar1* locus replacing the *BAR1* ORF, as part of an inserted plasmid with a *HIS3* marker. We removed this entire insertion by transforming SGA85 with a PCR product of a *∆bar1::URA3* locus that included 300 bp of promoter sequences and 700 bp of terminator sequences. We used strain GPY8190 as template for the *∆bar1::URA3* PCR. We selected for uracil prototrophs and confirmed appropriate gene replacement by the loss of the pheromone inducible CFP reporter, acquired histidine auxotrophy and by PCR. We named the resulting strain SGA114.

We then introduced a nuclear-localized fluorescent label by fusing the *HTB2* ORF to YFP. We used a PCR product containing the *YFP- T_ADH1_-KAN^R^(MX4)* cassette with ends with homology to sequences upstream and downstream of the *HTB2* ORF, that was generated with the *YFP-KAN^R^(MX4)* from the Pringle collection (Longtine et al., 1998a). We confirmed proper insertion by the appearance of a bright yellow fluorescence label in the nucleus and by PCR. We named the resulting strain SGA118.

We recovered the *URA3* marker by transforming SGA118 with a 200bp double stranded oligonucleotide composed of two 100 nt sequences with homology to the ends of the *URA3* marker in the *∆bar1::URA3* locus. We plated the transformation on 5-FOA medium, picked colonies that grew, and checked proper deletion of URA3 by PCR. We named the resulting strain GPY123.

We next made two derivative strains from GPY123, each carrying one gene deletion, for either *BIM1* or *GIM4*. We followed the same protocol described above for deleting *BIM1*, except that we used *HIS3(MX4)* as the selective marker. The resulting strains were named *GPY4144* *(∆bim1*) and *GPY4150* *(∆gim4*).

SEC8-RFP SPC42-GFP strains

This set of strains was derived from a clonal isolate from the O’Shea and Weissman GFP collection at UCSF (Huh et al., 2003). The members of this collection were derived from a BY4741 strain. The clone we used carried a modified allele of *SPC42* expressing Spc42 with a C-terminal GFP tag and linked to a *HIS3* marker (this allele is denoted here as *SPC42-GFP—HIS3*).

To make the reference strain for this set, we first deleted the *BAR1* gene in *SPC42-GFP—HIS3* using the approach we described above for the generation of GPY8190. This resulted in a *SPC42-GFP—HIS3* *∆bar1::∆marker* strain.

In a second step, we modified the *SEC8* gene to express Sec8 with a C-terminal mCherry tag. We used an approach identical to the one above for *P_PRM1_-YFP* and *-mRFP*, except that in this case we added the tag at the end of the gene instead of replacing the gene with it, and used the mCherry-T_ADH1_---G418^R^ template described before.

This resulted in strain GPY1751: *SPC42-GFP—HIS3 ∆bar1::∆marker SEC8-mCherry—KanMX6*.

In a final step to construct the base strain for this series, we replaced the *CDC28* gene with the analog-sensitive *cdc28-F88A* allele. We used linearized p*CDC28-as2-406* to “loop in” the allele, and then we “looped it out” as described (Colman-Lerner et al., 2005). We confirmed proper recombination by PCR and confirmed it by testing for 1-NM-PP1 sensitivity.

This resulted in strain *SPC42-GFP—HIS3 SEC8-mCherry—KanMX6* *∆bar1::∆marker cdc28-F88A,* named GPY1752. GPY1752 was the base strain for this set.

Starting with GPY1752, we generated GPY1709 (*∆bim1--Nat^R^MX6*) and GPY1710 (*∆gim4--Nat^R^MX6)* via the Pringle cassette approach described above for the GPY4000 series strains.

##### Construction of strains expressing human estrogen receptor chimeras

Vectors for homologous recombination with excisable CEN/ARS cassettes

For some cloning steps below we used the pTC41x series of derivatives of the pRS41x shuttle vectors (Gordon et al., 2007). Each pTC41x vector was made by cloning a CEN/ARS containing-fragment flanked by AatII overhangs into the AatII site of the cognate pRS40x vector. As a result all pTC41x vectors can be converted to pRS40x versions (“integrative” vectors that lack a yeast origin of replication) by cutting with AatII and religating.

Plasmid carrying an estrogen receptor chimera driven by the ADH1 promoter

Our source of the GAL4 DNA binding domain – human estrogen receptor – VP16 transactivation domain (GEV) chimera was plasmid pp1557, a gift of Peter Pryciak (Takahashi and Pryciak, 2008). We first subcloned a PvuI fragment of pp1557 containing *P_ADH1_-GEV-T_ADH1_* into the SmaI site of pTC415. To do this we co-transformed PvuI-cut pp1557 and SmaI-cut pTC415 into yeast strain BY4741. We isolated gap-repaired plasmids from leucine protrotrophs using a Zymoprep Yeast Plasmid Miniprep I kit. The resulting plasmids were then transformed into Z-competent *E.coli* to amplify the plasmid, 8 colonies were grown overnight in LB-Amp media and miniprepped using a Qiagen QIAprep Spin Miniprep Kit, and the plasmid sequences were confirmed by DNA sequencing. This cloning resulted in pSZ100.

We subsequently removed the CEN/ARS in pSZ100 by the AatII process described above, yielding pSZ101. pSZ101 elements are *P_ADH1_-GEV-T_ADH1_ ---LEU2*.

Plasmid carrying estrogen receptor chimera driven by the low variability BMH2 promoter

In a separate line of research we had found that the *ADH1* promoter showed unusually high cell-to-cell variability. For this reason, in parallel we constructed an alternative GEV expression vector driven by the *BMH2* promoter. We amplified the GEV coding sequence by PCR from pp1557, using primers that added to the amplicon ends with homologies to the 3’ end of the *BMH2* promoter (in the 5’ end of the amplicon) and to the pTC7 backbone (in the 3’ end of the amplicon, pTC7 is the plasmid from which pTC-*P_BMH2_-YFP-URA3* was derived (Colman-Lerner et al., 2005)). We cut pTC-*P_BMH2_-YFP- T_ADH1_-URA3* with EcoRI (partial digest) and XhoI to remove the YFP sequence. We co-transformed this digested plasmid with the GEV PCR product into yeast strain TCY3277, which carried a *P_GAL1_-GFP-T_ADH1_--HIS3* reporter. We transferred to *E. coli*, isolated plasmid DNA from transformed colonies, and mapped and sequenced plasmids that showed estradiol induction of GFP.

We named the resulting pTC-*P_BMH2_-GEV-T_ADH1_--URA3* construct pSZ110.

Subsequently, we moved the *P_BMH2_-GEV-T_ADH1_* cassette to the LEU2 vector pRS305. We cut pSZ110 and pRS305 with BglI and ligated both DNAs by standard cloning procedures. Recombinant plasmids were screened for those derived from a pRS305 backbone and containing the *P_BMH2_-GEV-T_ADH1_* cassette. The resulting plasmid was named pSZ111.

Strains carrying single copies of P_BMH2_-GEV and P_ADH1_-GEV

We designed both the *P_BMH2_-GEV* or *P_ADH1_-GEV LEU2* cassettes such that one or more copies could be integrated at the *LEU2* locus. To obtain strains with single copies of the GEV constructs, we first obtained a set of clones with unknown number of copies for each construct. To determine the number of GEV construct copies integrated in the clones we introduced pp1744 (a gift from Peter Pryciak containing *P_GAL1_-GFP-T_ADH1_ ---- His5_S. pombe_*), linearized at the *HIS3* locus with Nhe1, into each of them and screened the *P_GAL1_-GFP* transformants to identify those with just one copy of this GFP reporter. Finally, we stimulated the GEV clones with a gradient of estradiol doses and obtained GFP vs estradiol dose response. We use the dose responses to identify the clones with just one copy of the GEV constructs. For subsequent applications of these strains, we used the parents of these clones, because they did not carry the *P_GAL1_-GFP* construct. The steps we followed are described below.

We generated 8 clones carrying *P_BMH2_-GEV* by transforming pSZ111 linearized with XcmI at the LEU2 locus into GPY1804. 8 leucine protrotophs were saved and named GPY1806 (a-h). Similarly, we obtained 12 clones carrying *P_ADH1_-GEV* by linearizing pSZ101 with a PacI cut within *P_ADH1_* and transforming GPY1804. 8 leucine protrotophs were saved and named GPY1805(a-h). We then transformed all 16 strains, GPY1806(a-h) and GPY1805(a-h), with pp1744 linearized with Nhe1.

We determined how many copies of pp1744 were integrated in 2 isolates of each of the 16 transformations by measuring GFP levels after culturing cells for 3 hours in liquid medium containing 2% galactose. We selected 1 clone with one copy of pp1744 for each of the 8 clones each of GPY1806 and GPY1805 we had isolated earlier as described, and named them GPY1808 (a-h) and GPY1807 (a-h) respectively. Finally, we carried out estradiol dose – response curves in all 16 GEV clones carrying *P_GAL1_-GFP*. We grew GPY1808 (a-h) and GPY1807 (a-h) in glucose medium overnight to exponential phase and stimulated these cells at low density with a gradient of estradiol concentrations (1 to 50 nM range) for 3 hours. After 3 hours, we added 200µg/ml cycloheximde, incubated cells 5-9 hours at room temperature and measured GFP signal by flow cytometry. In each group of 8 clones, a majority of 5 to 7 clones showed identical dose responses. The remainder showed higher maximal induction and lower EC50. We judged that the dose responses shown by the majority of the clones corresponded to the single-copy integrants. From this point forward, we used the names GPY1805, GPY1806, GPY1807 and GPY1808 (without the lower-case letters sub-ID) were used to designate a single isolate of a single copy GEV integrant of each set.

The GEV locus driven by the BMH2 promoter is annotated as *BMH2::P_BMH2_-GAL4BD-hER-VP16--LEU2* in the strain table (Appendix Table S1).

Comparison of variability of activation dependent on P_BMH2_-GEV and P_ADH1_-GEV constructs

We first integrated a housekeeping fluorescent protein reporter in GPY1805 and GPY1806 by integrating pTC-*P_BMH2_-YFP-T_ADH1_—URA3* linearized with StuI within *P_BMH2_*. We identified strain with single-copy integrations and named them GPY1809 (*P_ADH1_*) and GPY1810 (*P_BMH2_*). Then we performed estradiol dose responses as above and analyzed the results as described earlier (Colman-Lerner et al., 2005). From this analysis, we found that the level of cell-to-cell variability in GEV activator function (amount and activity) in GPY1809 (*P_ADH1_*) was higher than in GPY1810 (*P_BMH2_*).

Based on these studies we decided to use GPY1810 (*P_BMH2_*) for subsequent constructions. At this point we decided to free the URA3 marker to allow the introduction of the *TUB1-828* construct (see below). We thus replaced the pTC-*P_BMH2_-YFP-T_ADH1_—URA3* integrant with a pTC-*P_BMH2_-YFP-T_ADH1_—MET15* integrant by loopout using 5-FOA selection followed by transformation with the MET15 plasmid linearized by cutting within *P_BMH2_.* We screened the transformants by fluorescence microscopy to identify single-copy P_BMH2_-YFP integrants. We called the resulting strain GPY1858. The locus is annotated as *BMH2::P_BMH2_-YFP--MET15* in the strain table (Appendix Table S1).

##### Construction of an estradiol inducible tub1-828-expressing strain (GPY1873)

We used an inducible *tub1-828* construct that we received from Kirk Anders (Anders and Botstein, 2001). This plasmid, pRB2949, is a CEN/ARS *URA3* yeast episome carrying a *P_GAL1_-TUB1-828* cassette. Expression of Tub1-828 protein from this construct is induced by galactose and repressed by glucose.

In our experimental system, incubation in medium without glucose with galactose alters many aspects of system behavior, including a substantial increase in pathway variability. We therefore induced *TUB1-828 expression* using the estradiol responsive Gal4-ER-VP16 chimeric transcription factor (described above,  Louvion et al., 1993).

We transformed GPY1858 with plasmid pRB2949 (Anders and Botstein, 2001). We tested several colonies for growth in the presence of estradiol. All of them failed to grow, the expected consequence of the induction of *TUB1-828.* We selected one clone for *TUB1-828* expression experiments and named it GPY1873.

After constructing GPY1873 and testing its effect on the pheromone response we found that basal *tub1-828* expression conferred by the estrogen-responsive chimeric transcription factor was enough to give the maximum response observed.

##### Construction of strains expressing ectopic PRS activating proteins for bypass experiments

If strains that contain artificial activator plasmids are grown on medium depleted of glucose, leak expression from *P_GAL1_* causes the artificial activator to activate the pheromone pathway and consequently cells arrest. For that reason, when using antibiotic selection to knock out genes, we first re-suspended these strains in 15ml of SDC medium (ie, medium that contained glucose) for 6 hours, spun down, re-suspended in 100μl, and plated directly onto antibiotic. This transformation protocol ensured that the cell cycle was not inhibited by expression of the ectopic activators prior to antibiotic selection.

In order to screen for single copy integration of activator plasmids, we used frozen stocks of 6 isolates to inoculate cultures into selective SDC medium. These cultures were allowed to grow for 6-8 hours and were then diluted to grow in log phase overnight. The following morning, we transferred these 6 cultures to medium that contained serial dilutions of β-Estradiol in SDC with 0.04 mg/ml casein and 10 µM 1-NM-PP1. These cultures were then fixed in 200µg/ml cycloheximde and taken to the flow cytometer for fluorescence measurements. Out of 6 isolates tested, the 4-5 that typically had the same dynamic range and maximum induction were scored as single integrations and given strain numbers.

P_GAL1_-STE4 activator strains

We transformed GPY1810 with pp1610 linearized with BsmI to target the plasmid to the *HIS3* locus and plated the mix onto histidine deficient media. We streaked 6 HIS+ isolates to single colonies, patched, and immediately froze them to prevent unwanted activation of the pheromone pathway. We named one isolate that was determined to have 1 copy of the plasmid, GPY1816. This strain was then transformed with the HygB^R^ PCR product mentioned above to knock out BIM1. 8 isolates were then screened by PCR for successful *BIM1* removal, and the *Δbim1* isolate was named GPY1818. Later, to suit experiments not shown here, we decided to free up *URA3* from these strains. To do so, we plated, GPY1816 and GPY1818 onto 5-FOA medium and collected single colonies, 4 of which were screened by PCR to verify the successful loop-out of the *P_BMH2_*-*YFP--URA3* plasmid. The resulting strains were named GPY1828 (*P_GAL1_-STE4*) and GPY1830 (*P_GAL1_-STE4* *∆bim1*) and transformed with a plasmid containing *P_BMH2_*-*YFP--MET15* linearized at the *BMH2* promoter with Stu1. 6 transformants of each were streaked to single colonies and those that carried single copy integrations were determined by microscopy, the resulting strains were named GPY1855 (*P_GAL1_-STE4*) and GPY1862 (*P_GAL1_-STE4 ∆bim1*). GPY1855 was then transformed with the HygB^R^ PCR product mentioned above to knock out *GIM4*; transformants were then PCR verified for the knock out. The resulting *P_GAL1_-STE4 ∆gim4* strain was named GPY1898.

P_GAL1_-STE5-CTM ∆ste5 strains

GPY1810 was transformed with pp1611 linearized with BssHI to target the plasmid to the *HIS3* locus and plated on histidine deficient media. 6 isolates were then streaked to singles, patched, and immediately frozen to prevent unwanted activation of the pheromone pathway. The isolate that was determined to have 1 copy of the plasmid integrated was named GPY1817. This strain was then transformed with the HygB^R^ PCR product mentioned above to knock out BIM1. 8 isolates were then screened for successful *BIM1* deletion, the positive isolate was named GPY1820. The same protocol was followed as for the *P_GAL1_-STE4* strain above to free up the *URA3* marker. The resulting strains that contained *P_BMH2_-YFP::MET15* instead of *P_BMH2_-YFP::URA3* were named GPY1856 (*P_GAL1_-STE5-CTM*) and GPY1864 (*P_GAL1_-STE5-CTM* *∆bim1*). GPY1856 and GPY1864 were then transformed with a NAT^R^ PCR product with tails homologous to the *STE5* promoter and terminator, to knock out *STE5* from the genome. Transformants were PCR verified for the deletions, positive isolates were named GPY1915 (*P_GAL1_-STE5*-CTM *∆ste5*) and GPY1916 (*P_GAL1_-STE*5-CTM *∆bim1 ∆ste5*). GPY1915 was then used to incorporate the *∆gim4* mutation as described for the *P_GAL1_-STE4* activator to generate strain GPY1997.

##### Construction of strains to quantify Ste5 membrane localization

YPP3662 (Ventura et al. 2014), which carries instead of Ste5 three copies of a *STE5-YFP-YFP-YFP* fusion gene, was the parental strain for these experiments. From it, we generated *Δbim1*, *Δgim4*, and *tub1-828-expressing* derivatives. We generated GPY4112 (*∆bim1*) and GPY4113 (*∆gim4*) via the same Pringle cassette approach using the URA3(MX4 plasmid) we used to make the GPY4000 series strains. We generated the otherwise-isogenic *tub1-828-expressing* strain by the same steps we used to generate the strains used in the bypass experiments: transformation by linearized pSZ111 followed by isolation of a *P_BMH2_-Gal4-hER-VP16* construct integrated at *BMH2*. We introduced into 8 transformed strains those strains a *P_Gal1_-mCherry* construct, and picked one strain from the majority of strains that showed low expression consistent with single copy insertion of the *P_BMH2_-Gal4-hER-VP16*. We called this strain ACL-GP-001. We then transformed it with pRB2949 (Anders and Botstein, 2001) which carries a P*_GAL1_-tub1-828* construct, to create GPY4121.

## **2 High-throughput screen and follow up studies**

### 2.1 Assembly of 96-format arrays of collection haploids isolated from single colonies

##### Selection of deletion strains included in the primary screen

Set 1: Unbiased Genes

This set consisted of 996 gene deletions randomly selected from the library. We made this set by picking clones from the library arrayed in 384 colonies format in order of appearance, starting in position A1, completing each row, and following with the next row. When a colony was missing we looked for a colony corresponding to the same gene deletion in the section for duplicates in the library, and added it if present. This selection was unbiased with respect to, among other things: gene location in the genome, gene ontology, name, ORF number and any phenotype of the deletion strain.

Set 2: Kinases and Phosphatases

To assemble this set we searched the Saccharomyces Genome Database (SGD) for genes annotated with “viable systematic deletion phenotype” and with the “function” GO terms “protein kinase activity” and “phosphoprotein phosphatase activity”. We retrieved 106 and 41 hits, respectively. Except for one gene in each set, all of these putative or confirmed protein kinases and phosphatases were represented in our modified gene deletion library. The total number of strains in this set was thus 145 (105 for protein kinases and 40 for phosphatases).

Appendix Table S2 contains the list of all strains screened.

###

### 2.2 High-throughput growth, pheromone stimulation and flow cytometry measurements

We stored at 4^o^C saturated cultures of 96-format clones from the modified deletion collection. To screen, we followed the procedure described below

##### Growth to exponential phase

#####

We used a slotted pinning tool to inoculate 5µl of the saturated cultures stored at 4^o^C into 500 µl of SDC media in 1.1 ml-capacity polypropylene 96-well plates. We grew these cultures to carbon exhaustion, 2 days at 30^o^C. We then inoculated 5µl of the freshly saturated cultures into 250 µl of SDC in 300 µl-capacity polycarbonate 96-well plates, grew them for 8-10 h at 30^o^C, and measured and saved the OD_600_ of all wells using a multiwell spectrophotometer. We used this measured OD information to calculate the dilution of inoculum needed to have most of the strains in exponential phase after 12-18 hours of growth. We then prepared several 300 µl-capacity polycarbonate plates as before and inoculated them using the 5 µl slotted pinning tool, at the calculated dilution, and 0.5, 1.5 and 3 times that amount.

##### Induction of the pheromone response system

After 15 h of growth, we measured OD600 of all plates and used the OD data to choose the plate in which the largest number of strains 1) was in exponential phase and 2) had enough cells to be used in the next step.

Immediately prior to stimulation, we sonicated the culture flat-bottom plates by “floating” the plates in the sonication bath of a S-3000 MP Misonix sonicator (Misonix Inc), set at power 10, for 2 minutes in two periods of 1 minute each with a 30 sec rest in between periods.

We next inoculated 5 µl of the sonicated cells into 250 µl of pheromone-containing medium in 300 µl-capacity polycarbonate 96-well plates. We followed the conditions described previously (Colman-Lerner et al., 2005). Briefly, stimulation media contained pheromone in SDC media containing 20 µg/ml caseine (SIGMA), to block pheromone binding to the plastic walls, 5-10 µM 1-NM-PP1, to inhibit Cdc28-as2 and 0.15 X strength PBS to buffer pH and thus prevent casein precipitation.

We incubated the cells in pheromone-containing medium for 3 h at 30 ºC for the screen and for most experiments, except when indicated. The 30 ºC incubation was done in an air heated ~30 cm rotation radius shaker at 250 rpm. After the end of the pheromone incubation period, we added 100 µg/ml cycloheximide to stop reporter accumulation and allow for complete fluorophore maturation. Yeast cells in cycloheximide retain their shape and external appearance for more than 10 hours at 30 ºC.

Finally, we sonicated the plates as described above and measured fluorescent protein expression by flow cytometry.

##### Flow cytometry measurements

We used a Becton-Dickinson LSRII flow cytometer equipped with a 100 mW 488 nm laser and a 150 mW 532 nm laser, as schematized in Appendix Figure S3. All filters and dichroic mirrors were from Chroma. We used a threshold value of forward scatter (FSC) from the 488 nm laser to trigger data collection. We calibrated threshold values of FSC to detect the smallest cells in an exponentially growing culture of wild type or mutant yeast cells. We measured YFP and mRFP (or mCherry) fluorescence from the light emitted during the 532 nm excitation, which was channeled, using mirrors, into an octagonal array of 8 photomultiplier tubes (PMTs), labeled A to H. Light entering the array was first split by a 735 nm long-pass dichroic mirror (735LP) and then split again by a 640 nm long-pass dichroic mirror (640LP). The light that came through the 640LP dichroic was filtered through a 675 nm band-pass filter of 50 nm wavelength width (675/50) before hitting the B PMT. The lower wavelength light that reflected from the 640 LP dichroic was directed towards a 600LP dichroic, and the reflected light from this was split by a 540LP dichroic. The light that passed the 540LP dichroic was filtered through a 550 nm band-pass of 10 nm width (550/10) before hitting the D PMT.

We took the signal from the B PMT as the fluorescence from mRFP (or mCherry). Cells expressing only YFP or CFP showed the same signal in this channel as wild type cells. We took the signal from PMT D as the fluorescence from YFP. Cells expressing only mRFP or CFP showed the same signal in this channel as wild type cells. We also took a signal for CFP, but this channel suffered from high background autofluorescence (largely from intracellular NAD(P)H UV-excited, cyan-emitting fluorescence) and was not useful for this project (we instead measured CFP expression by quantitative microscopy).

*2.3 Quantifying the different contributions to cell-to-cell variability in cell signaling and response.*

Since Delbrück, non-genetic non-environmental cell-to-cell variability has sometimes been attributed to statistical fluctuations in the output of biochemical processes, such as gene expression, that involve small numbers of protein components (Arkin et al., 1998; Delbrück, 1945; McAdams and Arkin, 1997). For example, Arkin and McAdams showed by modeling that stochastic fluctuations in gene expression could plausibly account for whether an infecting lambda phage lyses the cell or forms a lysogen (McAdams and Arkin, 1997). Such variability is often referred to as “noise” (Elowitz et al., 2002). This term can sometimes connote rapid fluctuations. However, other work reveals the importance of additional slowly-changing sources of variability in reducing coherence of population responses. In phage λ, incoming phage are more likely to lysogenize small cells than big ones, suggesting that one slow-changing source of variability (the cell growth and division cycle), rather than a fast-changing one (“noise” in the chemical reactions), causes the observed variability in the percentage of infecting phage that form lysogens (St-Pierre and Endy, 2008). Earlier work in *S. tymphimurium* showed that individual bacteria retain characteristic chemotactic behavior throughout its lifetime (Spudich and Koshland, 1976). Our work in the yeast pheromone response (Colman-Lerner et al., 2005, see below) revealed and quantified two slow-changing sources of variability, which we called L and G. Similarly, in mammalian cells, abundance of particular apoptosis regulators determines the different timing of apoptosis in individual cells (Spencer et al., 2009). The abundance of these regulators in sibling cells is similar and thus weakly heritable (Spencer et al., 2009). Such slow-changing cell-to-cell differences in protein abundance in cultured mammalian cells can, in some cases, predict drug response outcomes (Cohen et al., 2008).

In contrast, fast-changing fluctuations in protein concentration are unpredictable. For example, in the *lac* operon, variability in time to induction is caused by infrequent stochastic bursts in gene expression that arise from infrequent (in the order of once per generation) unbinding of the lacI repressor from its operators on the lac promoter (Choi et al., 2008).

Work by Elowitz et al. (Elowitz et al., 2002), measured expression of two different colored fluorescent proteins driven by different instances of the same artificial LacI (lac repressor) -regulated promoter (Lutz and Bujard, 1997) in populations of clonal *E. coli*. This work defined two quantities: “intrinsic noise", a measure of the extent to which the output of the two reporters did not correlate, and “extrinsic noise”, a measure of the remaining, correlated variability. Intrinsic noise, in most cases the smaller component, was presumed to arise from rapid-changing stochastic differences in the molecular events required for transcription and translation. Extrinsic noise was attributed to cell-to-cell fluctuations in the abundance of molecules such as regulatory proteins and polymerases. Both types of noise increased cell-to-cell variability in gene expression in the cell population.

*Analytic framework*

To dissect contributions to cell-to-cell variability in system output in the signaling arm of the yeast PRS, we used pairs of transcriptional reporters driven by different pairs of promoters (identical and non-identical, pheromone-inducible vs pheromone-insensitive). This experimental setup allowed us to separate cell-to-cell variability in molecular events upstream of the promoter (affecting a signal transmission or "pathway" subsystem, P) from those downstream (affecting an "expression" subsystem, E). It allowed us to further separate the contributions to variability in the gene expression subsystem caused by stochastic variability (γ) (Appendix Figure S4) from that caused by preexisting differences in the ability of cells to express proteins (G), and differences in signal transmitted by individual cells (P) (Appendix Figure S5). We quantified variability in gene expression due to stochastic gene expression noise, γ, by comparing outputs in each cell of genes carrying α-factor-responsive promoters driving the YFP and CFP reporter genes (Appendix Figure S4, drawn after Figure 1b in Colman-Lerner et al. 2005)). We then measure variability in pathway subsystem output (P) and expression capacity (E) in strains containing a pheromone responsive promoter driving YFP and a control promoter driving CFP reporter genes. (Appendix Figure S5, drawn after 1c in Colman-Lerner et al. (2005)). Here, different Pathway subsystems (blue boxes) regulate the activity of the DNA-bound transcription factors, but the subsystem enabling expression of the reporter genes (red box) is the same. Variability in expression capacity, G, affects the correlated variability (the dispersion of points along the diagonal. Uncorrelated variability (the dispersion of points along the minor axis) is due to the stochastic gene expression noise, γ, and to cell-to-cell variability in the pathway subsystems for each promoter. Although this analytical framework recognized contributions to differences in transmitted signal (P) caused by pre-existing differences in the ability of cells to send signals (L) and stochastic differences in the ability of cells to send signals during the course of the experiment (λ), the experiments above do not allow us to distinguish them experimentally.

We quantified total cell-to-cell variability using the normalized variance η^2^, the variance squared over the mean squared, σ^2^/μ^2^ (for G, P, E, and L, this is equivalent to the square of the coefficient of variation or CV). We also quantified three components of η^2^_total_: cell-to-cell variability in gene expression capacity, G, η^2^(G) (the overall capacity of a cell to transcribe genes into mRNAs and translate those mRNAs into proteins), “stochastic variability in gene expression” or "gene expression noise", η^2^(γ) (which corresponded to “intrinsic noise”), and “cell-to-cell variability in pathway subsystem output”, η^2^(P). Both η^2^(G) and η^2^(γ) reduced the coherence in population gene expression responses. In these experiments, we found the contribution of η^2^(γ) or intrinsic noise was very small. Even when we eliminated variability due to cell cycle position, most variability was due to differences in P and in G (Colman-Lerner et al., 2005). (As mentioned above, η^2^(P) is the sum of two components, which we could not separate experimentally: η^2^(L), and η^2^(λ)).

##### Derivation of formula for estimation of pathway variability

Here we present the derivation of the formula we use for pathway variability . In Colman-Lerner et al. 2005, we showed that the cell-to-cell variability observed in fluorescent reporter expression can be split into four contributions: (1) variability in signaling , (2) variability in gene expression capacity , (3) stochastic fluctuations in gene expression , and a correlation term , whereis the correlation between mean signaling capacity and mean gene expression capacity, computed over the population. In a case where we used an inducible yellow reporter and a constitutive cyan reporter, labeling the corresponding quantities with subscripts *y* and *c*, we would thus have

The measured correlation between these two reporters can also be split into contributions from the two subsystems:

In this work, we estimate from the data in the following way

where angle brackets indicate an average over the cell population of the enclosed quantity. Because the validity of this estimate may not be obvious at first, we derive it below:

Using the variance sum law, split the right-hand side of the previous equation into

From the definition of , and properties of covariance, this can be re-written as

Using the definition of , we can say

Inserting the definitions from Colman-Lerner et al 2005, as above, and performing the cancellations gives:

Since the constitutive pathway variability and the gene expression noise terms, i.e. the last three terms on the right-hand side, are all small (see supplement to Colman-Lerner et al 2005), this is a good way to estimate the inducible pathway variability.

The neglected terms are all positive, thus the computed quantity (the left-hand side) represents an upper limit for , i.e.

To summarize, *y* and *c* are values of the total fluorescence (two different colors) from each cell in an isogenic population. The variance of the difference between *y* and *c*, both normalized by their respective means, is a measure of the uncorrelated variability visible on a scatter plot of *y* vs. *c*. If both *y* and *c* are driven by constitutive promoters, then this is in turn a measure of , i.e. stochastic noise in gene expression. If, on the other hand, y is driven by an induced promoter and *c* by a constitutive promoter, then we know from previous work (Colman-Lerner et al., 2005) that the variance of the difference between the normalized fluorescences includes a much larger contribution from . We can thus use this variance as an estimate of the cell-to-cell variability in transmitted signal, .

*Computing the progressive spread distribution (PSD) and the median progressive spread (MPS)*

To quantify the perception that in microscopic time course measurements, the spread of trajectories for cells of some strains differed in both magnitude and quality from the corresponding spread for other strains, we developed a statistical measure we called the progressive spread distribution (PSD). For each strain, we computed the PSD by first computing the absolute deviation of each cell's final pathway output from the median. We then sorted these deviations from smallest to largest. We found the first value of the PSD by starting with the two trajectories with smallest absolute deviations and finding the difference between the corresponding pathway output values. We computed the next value of progressive spread by including the value of pathway output corresponding to the cell trajectory with the third smallest deviation, and again finding the range of corresponding outputs, and so on until we had included trajectories for all the cells in each tested population of each strain. For a given set of pathway output measurements, the last value of the PSD was simply the full range of the pathway output values. For symmetric distributions, the median of the PSD, which we called Median Progressive Spread (or MPS), is equivalent to the interquartile range.

*Quantifying the stability of individual time course trajectories: the crookedness index I_C_*

For each cell, at 35-40 minute intervals, we recorded total fluorescence from a pheromone-inducible reporter and from a constitutive reporter. (For cells of the wild-type and Δbim1 strains, we measured six times; for Δgim4 cells, we skipped the first of these measurements.) In main text Figure 4, these reporters were *P_PRM1_-mCherry* and *P_BMH2_-YFP,* respectively. We divided, pointwise, the mCherry total fluorescence by the YFP total fluorescence to obtain a cell-size-invariant measure of each cell’s response as a function of time. This measure is our estimator of the transmitted signal P. For each of the five available consecutive pairs of measurement times (four pairs for *Δgim4*), we computed the average time rate of change of P, i.e. we subtracted the next value of P from the current one, and divided by the time between measurements. For each individual cell, we also fitted a linear model to its entire time series of P. We used the slope values from these linear fits to normalize the time rates of change; thus a cell with a perfectly stable response would have normalized time rates of change all equal to 1. We thus subtracted 1 from the normalized time rates of change, and took the absolute value, to obtain a measure of the instability of each cell’s response during each measurement interval. We then computed the average over time intervals to obtain an overall stability measure for each cell’s trajectory; this is the measure we named “crookedness”. Crookedness is always positive or zero; zero indicates a perfectly stable trajectory.

### 2.4 Selection of candidates for further screening, secondary and tertiary screens

##### **2.4.1 Selection of candidates from primary screen**

We used the high-throughput growth, stimulation and flow cytometry process described above to screen two sets of gene deletion mutants from the deletion collection generated.

We selected gene deletion strains for follow up studies based on their pathway variability and pathway output values as described in the main text. We used as reference the values obtained from the included unmodified reference strains (SGA85, present in 4 wells in each plate) and the overall distribution of values for all the strains, both deletions and controls. Based on these two criteria we defined thresholds to select the mutants (main text Figures 2a, 2b, and 2c) so that the mutants and approximately 10% of the SGA85 controls had values outside the selected thresholds. We thus selected from each set.

Set 1 (unbiased genes): 102 of 996 gene deletion strains.

Set 2 (kinases and phosphatases): 38 of 145 gene deletion strains.

##### **2.4.2 Secondary screens**

Repeated assays on the same independently isolated segregants

We included this step only for the gene deletions selected in Set 1.

We consolidated all 102 strains selected by their variability (see above) in 2 new culture plates with fresh medium, grew them to saturation and assayed as described above for the primary screen.

95 of the 102 re-tested deletion strains again showed change above or below the thresholds and were selected for the next round of follow up.

Assays on three independently isolated segregants.

We applied this next step to all Set 1 candidates that showed reproducible results in the repetition above and to all Set 2 candidates.

Given that we had set thresholds so that approximately 10% of the SGA85 strains would also be selected, we expected that approximately 10% of the selected genes would not show a reproducible difference from reference when re-assayed.

We assayed three new segregants from the same haploid selection plates on which the sporulated SGA88 x deletion strain diploid had been streaked. We picked three new colonies and inoculated them in fresh medium in 96-well culture plates. We applied the same criteria as in the first pick, selecting colonies of representative size and avoiding colonies that were unusually large. For each deletion strain we grew four cultures in these new plates: three cultures from the newly isolated segregants and a fourth culture inoculated from the previous culture grown for the primary and first secondary screens. Each plate also included 4 to 6 wells with SGA85 cultures (reference strains).

We found the following number of strains that passed this test (cell to cell variability in signal transmission η^2^(P), or total system output from the pheromone-inducible reporter were above or below the distribution of values of the reference strain cultures):

Set 1 - 37 of 95

Set 2 – 17 of 38

Total – 50 of 133

We confirmed by PCR that all these strains carried the gene deletions attributed to them.

Description of the 50 gene deletions that yielded reproducible results is shown in main text Table 2, a heat map of the outputs of their reporters in main text Figure 3, and those values in Appendix Tables S3 and S4.

**2.4.3 Tertiary screen: Microscope cytometry assays for cell-cell variability**

We performed a follow up assay using fluorescence microscopy. This assay allowed us the CFP fluorescence signal accurately, which we could not accomplish using our flow cytometry setup. In our gene deletion library CFP is driven by *P_PRM1_*integrated at the *BAR1* locus; *P_PRM1_* also drives mRFP at the *PRM1* locus. With this pair of reporters we could measure gene expression noise η^2^(γ) from the *P_PRM1_* (Colman-Lerner et al., 2005). In addition to providing gene expression noise values, the microscopy assays allowed us to verify that any increase in pathway variability (η^2^(P))was in fact due to differences in reporter-derived cytosolic fluorescence between isolated, live cells and not a secondary consequence of cell aggregation, unusual cell shape, autofluorescence specks or other interfering factors.

We measured by microscopy outputs from strains of interest, including the 44 listed below in Appendix Table S5 plus reference strains. Appendix Table S6 shows the results.

| Appendix Table S5. Strains from flow screens tested by microscope cytometry. | | | | | | | |
| --- | --- | --- | --- | --- | --- | --- | --- |
| Unbiased screen | | | | **Kinase and phosphatase screen** | | | |
| 1 | *MSH1* | 12 | *RPL34A* | 1 | *BUB1* | 12 | *OCA1* |
| 2 | *ARG82* | 13 | *RPP2B* | 2 | *CLA4* | 13 | *PBS2* |
| 3 | *BIM1* | 14 | *SLA1* | 3 | *CKB2* | 14 | *PKH1* |
| 4 | *DEP1* | 15 | *SUM1* | 4 | *CTK1* | 15 | *PPG1* |
| 5 | *ERD1* | 16 | *SWI5* | 5 | *ELM1* | 16 | *PPZ1* |
| 6 | *FUS1* | 17 | *SXM1* | 6 | *FUS3* | 17 | *PRR1* |
| 7 | *KAR4* | 18 | *UBA4* | 7 | *GAL83* | 18 | *RCK1* |
| 8 | *MKC7* | 19 | *UME6* | 8 | *HOG1* | 19 | *SAP155* |
| 9 | *PAC10* | 20 | *VPS64* | 9 | *HSL1* | 20 | *SKY1* |
| 10 | *RPL12A* | 21 | *YHR189W* | 10 | *KIN3* | 21 | *SSK2* |
| 11 | *RPL19B* | 22 | *YKL137W* | 11 | *KSS1* | 22 | *YVH1* |

Protocol for microscopy assay

We grew cells in test tubes in SDC medium in two phases. First, the strains were inoculated into SDC medium from single colonies on petri dishes streaked no more than 10 days earlier from a frozen stock, then cultured for 6-10 h at 30 ºC. These pre-cultures were diluted into new test tubes with SDC, at different densities, calculated based on the known doubling time of each strain, and cultured for 15-20 h at 30 ºC. Cultures with OD_600_ between 0.2 and 1 were used for the microscope assay.

We sonicated cells in Eppendorf tubes and incubated them for 3 hours at 30 ºC in media with 10 μM 1-NM-PP1 and 0, 0.6 nM or 20 nM α-factor. Cell densities in the assays were in the OD_600_ 0.02 – 0.1 range. At end of the 3 h incubation cycloheximide was added to a final concentration of 100 µg/ml and samples were incubated at 30ºC for 5-7 h. Cell images were obtained and analyzed as described previously (Colman-Lerner et al., 2005; Gordon et al., 2007).

### 2.5 Follow up studies of effect of gene deletions and other perturbations

##### Dose-response assays

For all dose-response assays on the gene deletions of interest and/or all other perturbation experiments, we adapted the high throughput protocol we used for the screen. The only modification was in how we cultured the cells. Instead of culturing them in deep-well 96-well plates, we used the test tube protocol described above for the fluorescence microscopy secondary screen. We did the pheromone stimulation step exactly as in the assays we used in the screen, in shallow-well 96-well plates, with the only difference being the addition of pheromone, which in this case was done using a pipette from a serial dilution of pheromone stocks instead of the slotted-pin tool we used for the screen. All dose response experiments were measured by flow cytometry.

##### Consideration of morphology of Δbim1 and Δgim4 strains

We verified by light microscopic observation that, compared with reference cells, all *∆bim1* and *∆gim4* strains, including those in the arrayed collection and the deletions made freshly in the GPY4000 background, showed no morphological differences, during normal growth or after treatment with stimulating pheromone concentrations. We assessed the morphology of these mutants in microscopic assays of cell to cell variability. In addition, when we performed the Ste5 signaling patch formation experiments in main text Figure 8, we again verified by eye that those strains showed no morphological differences to the reference strains. We also examined a published repository of images of cells from the original haploid deletion collection (Ohya et al., 2005), http://yeast.gi.k.u-tokyo.ac.jp)) and confirmed that *Δbim1* and *Δgim4* cells from the original deletion collection did not show morphological differences from the reference B4741 strain.

##### Chemical disruption of microtubule polymerization

One of the means we used to perturb microtubule formation was growth in medium with high concentrations of nocodazole (10 µg/ml) and benomyl (30 µg/ml), two chemically distinct but functionally equivalent inhibitors of microtubule polymerization. To prepare the medium with these drugs we first heated to 100^o^C a solution containing all ingredients for SDC except glucose and buffered with low-strength PBS (0.15 times the standard concentration). We then added 30 µg/ml benomyl from a 10 mg/ml stock in DMSO made daily. We added the same volume of DMSO to other control cell cultures. We then allowed both sets of liquid to cool to 50^o^C, at which point we added 20 g/L glucose and 40 µg/ml casein. Finally, we added 10 µg/ml nocodazole from a 10 mg/ml DMSO stock stored in small aliquots at -20 ºC. We added the same volume of DMSO to the control media. Finally, when indicated, we added 1-NMPP1 was added to a final concentration of 10 µM.

We tested each batch of nocodazole/benomyl medium for its effectiveness at inducing cell cycle arrest in a reference strain. To do so we grew a culture of BY4741 to log phase, spun the cells and resuspended the pellet in the nocodazole/benomyl medium. After a 3-hour incubation, we counted the number of cells arrested in metaphase, judged by their large budded appearance. The medium was considered effective if the fraction of arrested cells was equal or larger than 0.95.

##### Analysis of correlations between nucleus to signaling site distances and pathway output

Effects of microtubule perturbations on the position of the nucleus relative to the signaling site

To pursue the question of whether variability in nuclear position caused variability in transmitted signal, we determined the relation between P and the distance to the nucleus from the signaling site in single cells. In *∆bim1* cells, Bloom and collaborators had measured this distance under saturating pheromone stimulation (Maddox et al., 1999). Based on this and other work (Maddox et al., 2003; Zaichick et al., 2009) we expected that *∆bim1* cells would not be able to properly position their nuclei during pheromone stimulation. However, there was no published work on the effects of the *∆gim4* mutation or other prefoldin mutants on nuclear positioning. Thus, as a next step we sought to learn the effects of microtubule perturbations on nuclear positioning.

To better measure the position of the nucleus, we made a set of strains whose reference strain was GPY1752 (Appendix Table S1). In this set (Appendix Figure S6, Panel A i-iv) we expressed an Spc42-GFP fusion protein, which labels the SPB (Spindle Pole Body, the yeast MTOC, MicroTubule Organizing Center), and a Sec8-mCherry fusion protein, which labels the signaling site/ polarity patch. We then stimulated these cells with pheromone. The use of these two labels allowed us to obtain precise measures of the distance between the SPB and the signaling site at all pheromone doses. Appendix Figure S6 shows this distance information at two doses, saturating (Panel A ii) and EC_50_ (Panel A iv). However, because the signals were weak and we needed to acquire several images in the z-axis to find them, photobleaching of the fluorophores prevented us from taking time-courses in individual cells. Appendix Figure S6, A, i shows results two hours after pheromone addition. In these strains at saturating pheromone doses, the Spc42-GFP signal appeared as a single dot in the cytosol of each cell, and the Sec8-mCherry signal formed a small crescent at the shmoo tip (Appendix Figure S6, A, i). At concentrations near the receptor EC_50_, Sec8-mCherry localized to one of the two otherwise indistinguishable ends of the enlarged arrested cells (Appendix Figure S6, A, ii). Appendix Figure S6, A, ii and Appendix figure S6, A, iv show the distance between nucleus and signaling site. In reference cells stimulated at saturating pheromone doses, the nuclei were between 1 to 3 µm from the signaling site (Appendix Figure S6, A, ii), in agreement with the work of Maddox et al. (1999). In *∆bim1* cells the mean SPB-to-signaling site was larger, and the distance ranged from between 2 to 7 µm. In *∆gim4* cells, the mean SPB-to-site distance was unchanged relative to the reference cells, but the distribution of distances was broader. In reference cells at the EC_50_ pheromone dose, which causes cell cycle arrest but not shmooing (Appendix Figure S6, A, iii), we observed an even shorter distance between the nucleus and the signaling site, 0 (too close to resolve) - 2 µm, including a striking ~30% of cells in which we could not measure any distance between the nucleus and signaling site (Appendix Figure S6, A, iv). At the EC_50_ dose, effects of the *∆gim4* and *∆bim1* mutations on nuclear distance were qualitatively similar to those at saturating pheromone, with the exception that the *∆gim4* cells showed larger SPB to nuclei distances, in the 1-3 µm range.

We then measured the position of the nucleus in the same cells in which we analyzed PRS output trajectories. To do so, we used strains derived from GPY123 (Appendix Table S1). These strains expressed Htb2-YFP, which labeled the nucleus, allowing us to measure the shortest distance between the base of the mating protrusion (starting with the site on the cell perimeter at which the protrusion starts forming) and the nuclear edge. We first plotted time courses of nuclear distance from the shmoo base in these cells, stimulated with a saturating dose of pheromone. Appendix Figure S6, shows the results. As expected and described (Maddox et al, 1999), in the reference cells nuclei moved synchronously and rapidly from various initial locations to 0-1 µm away from the base of the mating protrusion. In *∆bim1* populations some nuclei moved towards the base of the mating protrusion, while other nuclei seemed to wander in the cytosol. In *∆bim1* cells, both sorts of nuclear movement were characterized by sudden changes in position, more frequent and drastic than in reference cells. In contrast, in *∆gim4* populations nuclear position was as coherent as in reference cells. However, in *∆gim4* cells, nuclei took longer to initiate their movement towards the shmoo base, with the majority remaining at the same distance for two hours or more.

Taken together, these results were consistent with the idea that microtubules comprise a tether connecting the signaling site and the nucleus that helps the nucleus hold position. They were also consistent with the idea that the *∆bim1* and *∆gim4* mutations partially disrupt its function. They confirmed previous observations about Bim1’s role in positioning the nucleus during the pheromone response and showed that prefoldin activity is also required for this process. Our results demonstrated that both mutations causing increases in η^2^(P) also caused changes in one or more aspects of the position of the nucleus relative to the signaling site, including average distance, variability in distance, and tempo of positioning.

Analysis of correlation between nuclear position and pathway output

We next sought to test the hypothesis that the amount of transmitted signal depended on nuclear position. If so, in single cells, output O, which depends on P, might be negatively (or positively) related to the distance between the signaling site and the Htb2-YFP labeled nucleus. To test the relationship between distance and P, we generated a *kar3-1* (rigor mutation) Htb2-YFP strain in which the microtubule bridge was stable and did not change in length. The Kar3-1 protein binds microtubule plus-ends, but can neither actively depolymerize nor release them. *kar3-1* strains therefore maintain cytoplasmic microtubules attached both the shmoo tip and nucleus, and these microtubules do not change in length (Maddox et al., 2003; Meluh and Rose, 1990). In fact, in our *kar3-1* strain, the nucleus did not move to the shmoo base on induction, its distance from the signaling site varied significantly from cell to cell (1.5 to 6 µ, Appendix Figure S6, C), and that distance did not change over time. Appendix Figure S6 C shows no correlation between nucleus to signaling site distance and pathway P in this strain. This result was inconsistent with the idea that P was a function of distance between nucleus and the signaling site, and showed that the MAPK signal can reach the nucleus even when the nucleus has not moved to the base of the shmoo tip.

*Cross stimulatory interactions and positive feedbacks at the signaling site*

One can imagine scenarios by which changes to microtubule abundance, structure, and function might cause variability in signaling. The molecular interactions shown in Figure 9 in the main text illustrate one. That figure shows a positive feedback mechanism by which small irregularities in Ste5 recruitment or Fus3 signaling might become amplified into larger differences in Ste5- or Fus3-dependent signaling: disruption of plus-end function of microtubules near the site might cause differences in delivery of signaling components; such differences would then become amplified. A second, similar way that irregularities in Fus3 signaling might become amplified depends on the fact that Gpa1 activates the RNA binding protein Scp160. Scp160 complexes with mRNAs encoding polarity and signaling proteins (called POLs), including the mRNA coding for Fus3 (Gelin-Licht et al., 2012). These mRNAs are trafficked down actin cables to the site, and, when translated, increase the amounts of proteins at the site, including Fus3. Activated Fus3 protein then sends signal in the PRS and stimulates the formation of still more actin cables as in Figure 9. A third amplifying mechanism is a well-studied positive feedback (Appendix Figure S7) involving Cdc42. Activated (GTP-bound) Cdc42 recruits a complex containing Bem1, Cdc24, and Ste20, the PRS MAPKKKK. Membrane associated Cdc24 (a guanine exchange factor or GEF) activates additional membrane-bound Cdc42. Activated Cdc42 recruits additional Bem1, Cdc24, Cdc42, and Ste20 (Johnson et al., 2011; Kozubowski et al., 2008).

**3. Table and Figure legends**

**Appendix Table S1 Strain table.** Genotypes of strains used in this study.

**Appendix Table S2**. List of strains screened via flow cytometry, with numerical data for expression-related quantities.

**
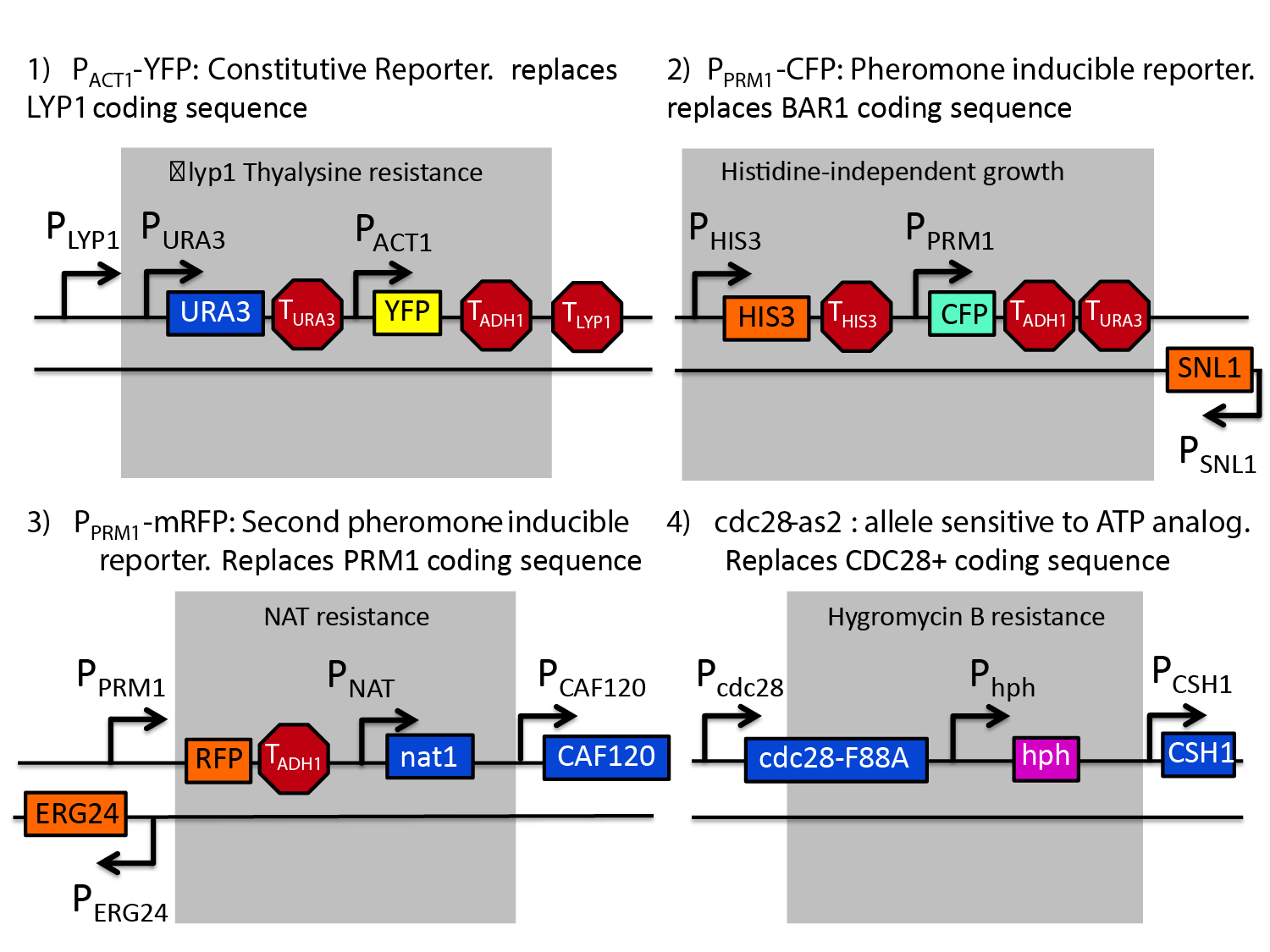
Appendix Figure S1**. **Key markers in strains in the reporter collection. (1)** *P_ACT1_-YFP*constitutive reporter. Replaces *LYP1* coding sequence. The *LYP1* deletion allows counterselection of diploids using L-thialysine. **(2)** *P_PRM1_-CFP*, pheromone-inducible reporter, replaces *BAR1* coding sequence. (3) *P_PRM1_-mRFP*, a second inducible reporter, replaces the *PRM1* coding sequence. (4) *cdc28-as2* allele installed in place of *CDC28* coding sequence. Confers sensitivity to the ATP analogue 1-NM-PP1, preventing cell cycle inhibition of the pheromone pathway and causing cell cycle arrest in G2/M.


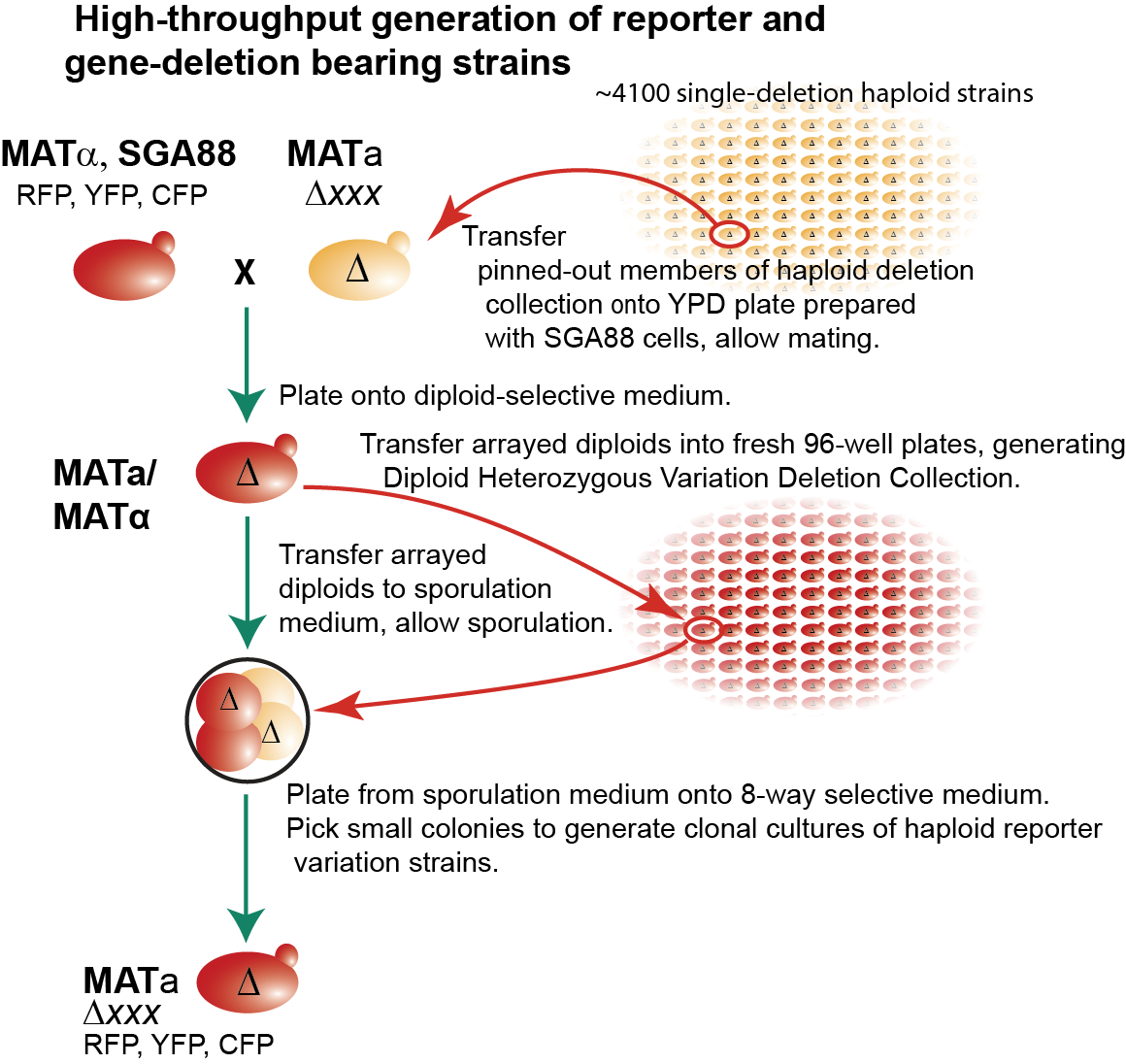


**Appendix Figure S2.** **High throughput generation of reporter and gene-deletion bearing strains.** We crossed the BY4742 derivative SGA88b (*MATα* *Δcan1::P_MFA1_-LEU2* *Δbar1-orf::P_PRM1_-CFP--HIS3* *∆bar1-promoter::ura3-terminator* *∆lyp1::P****_ACT1_****-YFP--URA3 ∆prm1::P_PRM1_-RFP--NAT(MX4) cdc28-F88A--hph(HygB^r^)(MX4)*) to strains of the yeast *MATa* haploid deletion collection (*Δxxx::G418 (MX6)*)where xxx is the deleted yeast gene) using 384 pinning tools. We sporulated the resulting diploids, and selected spores of the desired genetic makeup: *MATa* *Δxxx* *Δcan1::P_MFA1_-LEU2* *Δbar1*-*orf::P_PRM1_-CFP--HIS3* *∆bar1-promoter::ura3-terminator* *∆lyp1::P_ACT1_-YFP--URA3 ∆prm1::P_PRM1_-RFP—NAT(MX4) cdc28-F88A--(hph(HygB^r^)(MX4))*, grew these into single colonies and re-streaked these to obtain the haploid collection used in this screen.


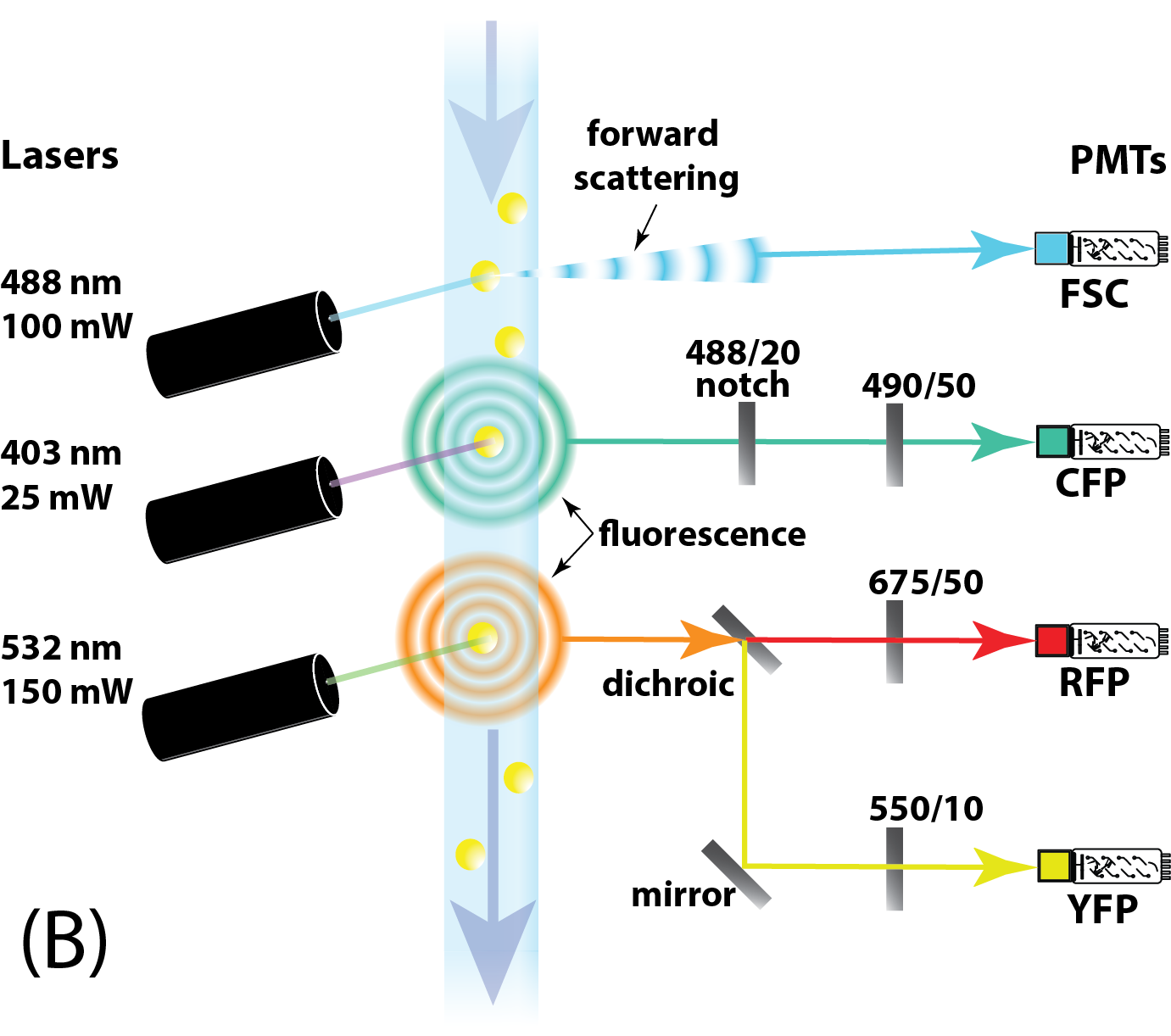
**Appendix Figure S3**. **Light path enabling three-color flow cytometric genetic screen**. Forward-scattering of 488 nm laser light indicates the presence of cells (yellow), while fluorescence in three separate wavelength bands indicates the quantity of cyan, red, and yellow fluorescentreporterproteins within cells. Photomultiplier tubes detected scattering and fluorescence intensities and recorded these in channels correspondingly labeled FSC, CFP, RFP, and YFP. Barrier bandpass filters, each labeled above with the center wavelength of the passband and the bandwidth in nanometers, ensured that excess laser light did not enter the PMTs. An additional bandstop filter (“notch”), labeled with the center wavelength and bandwidth of the stop band, ensured that light from the 488 nm laser did not enter the CFP PMT.

**
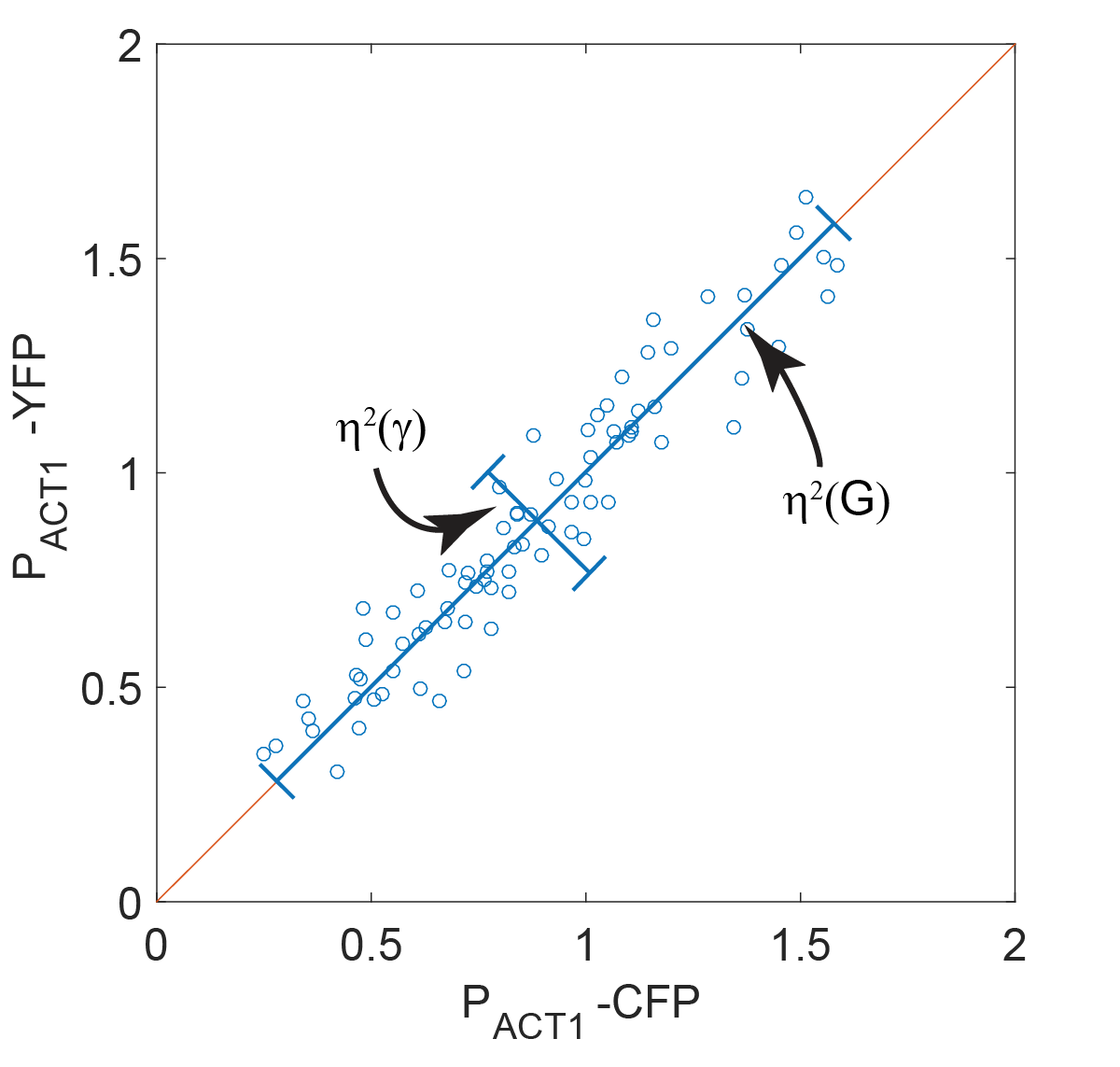
Appendix Figure S4**. **Measurement variability in gene expression capacity η^2^(G) and gene expression noise γ ** In this illustrative example, measurement depends on quantification in each cell of YFP and CFP reporter genes each driven by a different instance of the same constitutive *P_ACT1_* promoter. The spread of points along the correlation line shows cell-to-cell differences in the general ability of cells to express genes into proteins (η^2^G, while the spread of points across the correlation line corresponds to stochastic variability during the time of the experiment in the molecular processes needed for gene expression (η^2^ .

**
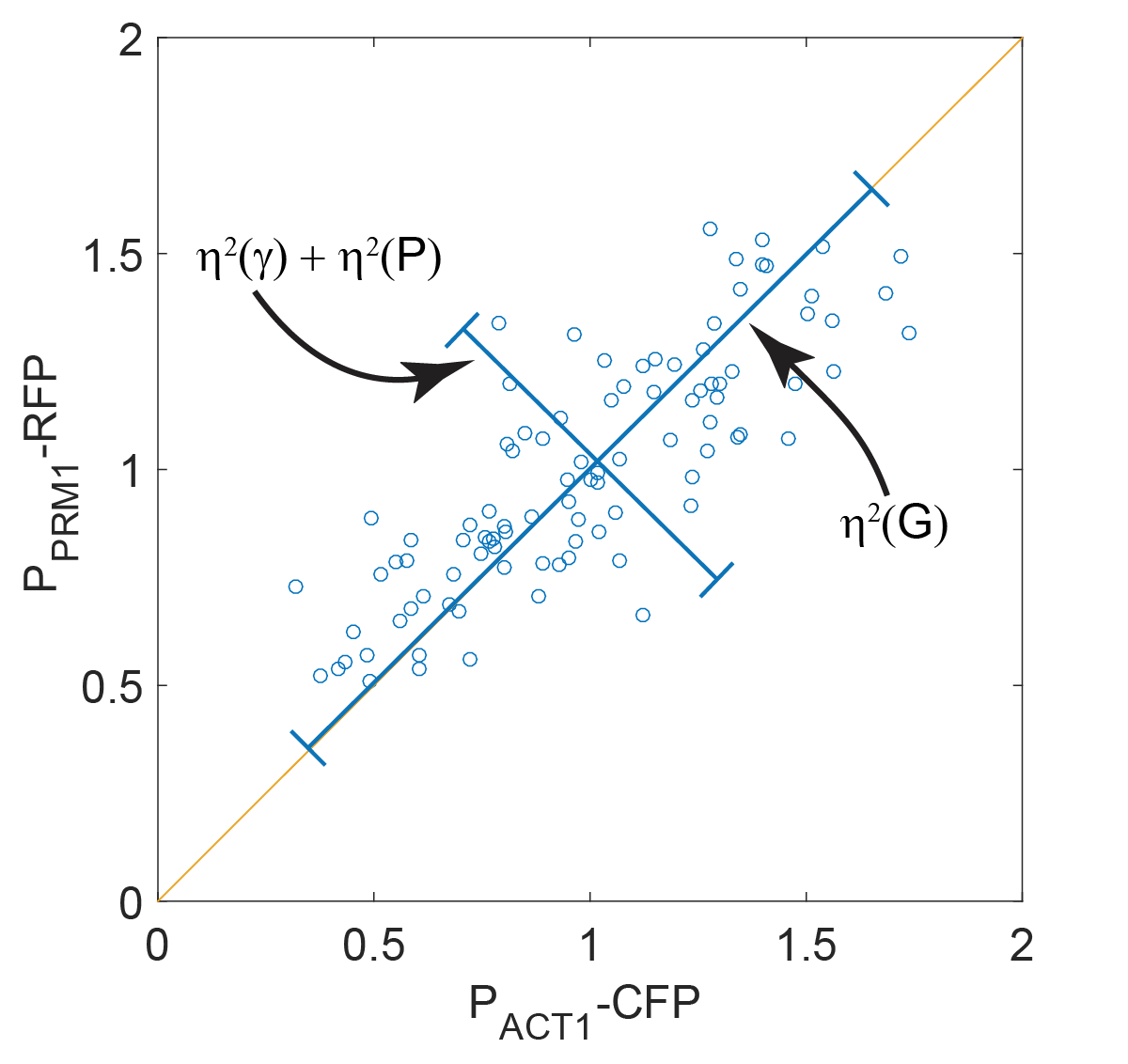
Appendix Figure S5**. **Measurement of pathway variability η^2^(P)**. Strains used in this figure carry a pheromone responsive promoter *P_PRM1_* driving RFP and a second reporter in which a control, constitutive *P_ACT1_* promoter drives CFP. Within a given cell, the Expression subsystem (transcription and translation) is the same, but the activity of *P_PRM1_* also depends on the accumulated signal transmitted to the promoter by the PRS. Expression of the two reporters is correlated (see the dispersion of points along the main diagonal), due to cell-to-cell differences in gene expression capacity G. Uncorrelated variability (the dispersion of points along the minor axis) is due to the combined effects of stochastic gene expression noise γ and of cell-to-cell variability in transmitted signal P of the inducible *P_PRM1_*. Because there is only one instance of *P_PRM1_*in these cells, and determination of γ for *P_PRM1_* would require use of two different P_PRM1_ reporters as in Appendix Figure S4, experiments like the one shown here do not allow us to measure γ They do however allow us to compute η^2^(P) + η^2^(γ), and since η^2^(γ) is much smaller than η^2^(P), we use that as an estimate for η^2^(P) for each cell as described in in this Appendix. They cannot, however distinguish between the components of variability in P, i.e. variability in L (the pre-existing component of P, differences in the ability of cells to send signal through the PRS), and the stochastic component, λ, due for example to stochastic differences in molecular signaling events during the course of the experiment.

**
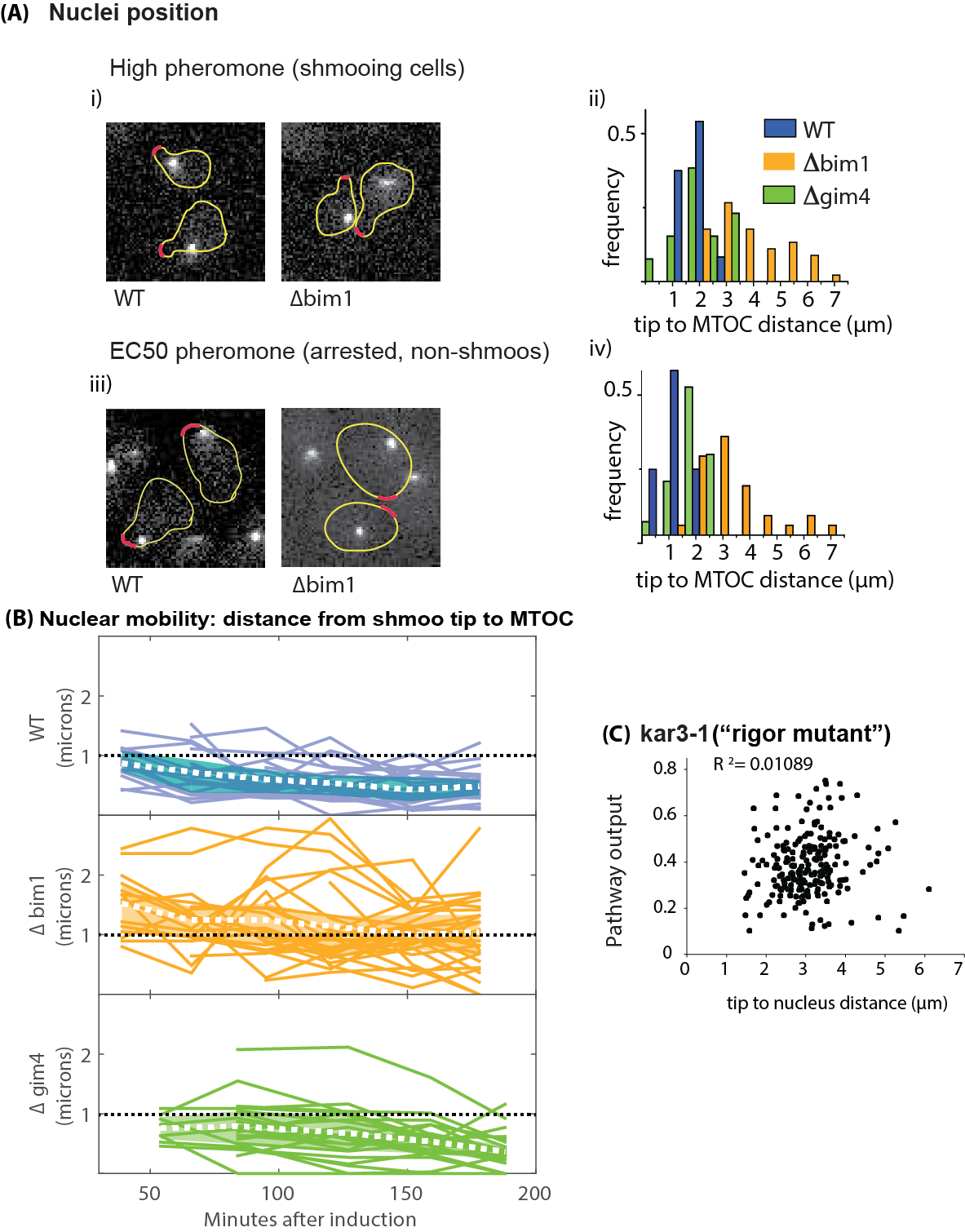
Appendix Figure S6. Distance and change in distance between nucleus and signaling site in microtubule perturbed cells (A)** Images of cells expressing Spc42-GFP, which marks the MTOC, and Sec8-RFP, which marks the signaling site. On the left, images show reference ("WT") and Δ*bim1* cells (GPY1752 and GPY1709)-- cells of the *Δgim4* strain (GPY1759) were visually indistinguishable from images of WT cells and thus are not shown. Images were recorded after 90 min of exposure to high (20 nM, i-ii) or intermediate (2 nM, iii-iv) pheromone doses. On the right, histograms show distances in μm between the MTOC and the signaling site (the shmoo tip) for reference (WT, blue), Δ*bim1* (orange) and Δ*gim4* (green) cells. **(B)** Time courses of the distance between base of the shmoo (where the cell wall is before the protrusion starts growing) and the nuclear edge (visualized by Htb2-Venus) after stimulation with 20 nM pheromone, for reference, *Δbim1*, and *Δgim4* cells as in A, with the same color-coding. At each time point, we computed the 0.25 and 0.75 quartiles, and used these to shade the middle two quartiles. The white dashed line marks the mean, and we drew a dashed black reference line to indicate 1 μm distance. **(C)** Signaling strength (P) vs. shmoo tip-to-nucleus distance in *kar3-1* (rigor mutant) strains. We stimulated *kar3-1* cells (SGA150, expressing Htb2-Venus to mark the nucleus and bearing *P_PRM1_-mCherry* and P*_ACT1_-CFP* reporters to monitor P) with 20 nM pheromone for 180 min. Panel shows P vs distance between the site of polarization and the nucleus for *kar3-1* cells, with each dot representing a single individual cell from this clonal population. We estimated Pathway output P from the ratio between the output of the *P_PRM1_* and *P_BMH2_* reporters. At this pheromone concentration, these *kar3-1* populations showed three-fold higher pathway variability (not shown).

**
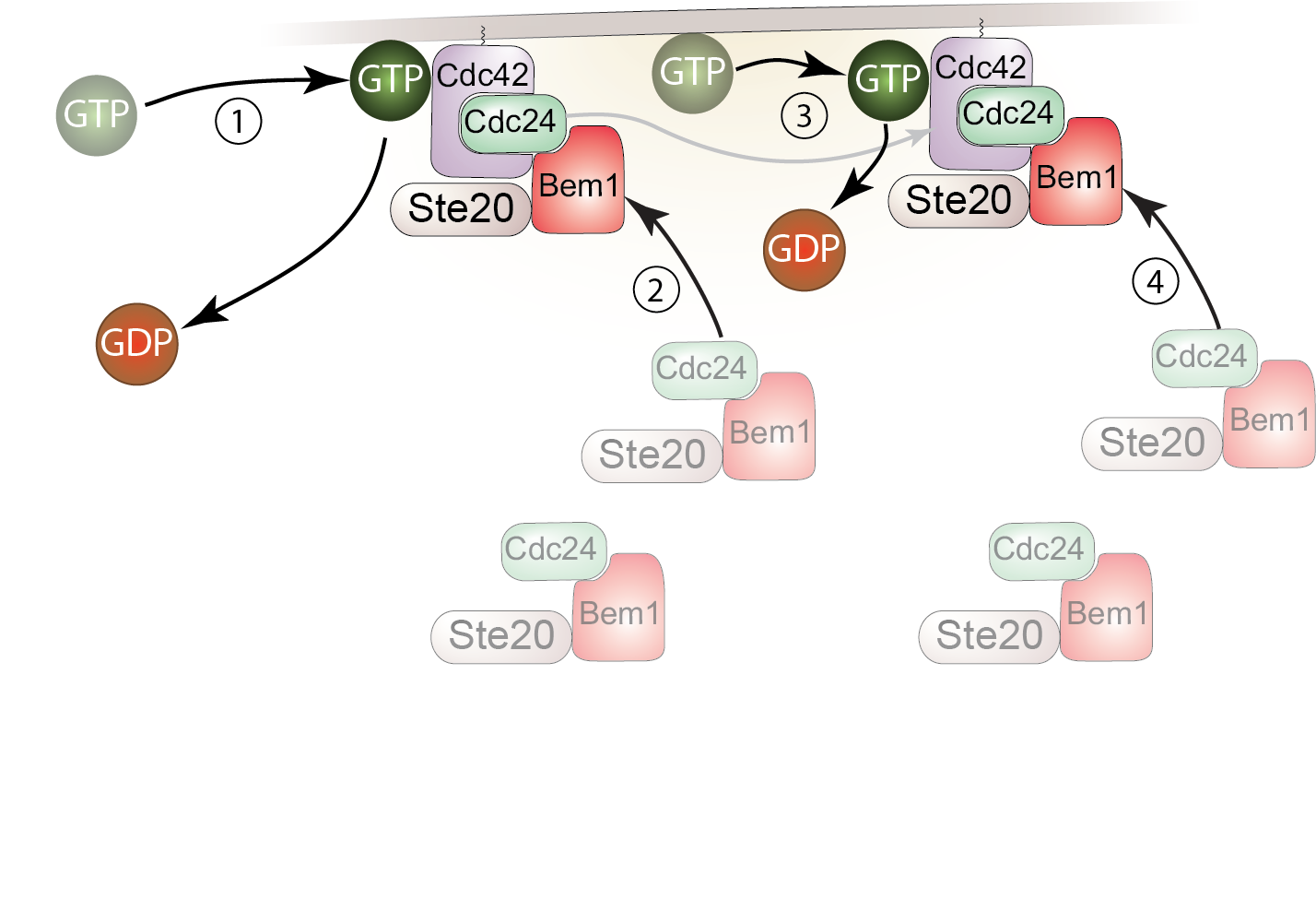
Appendix Figure S7**. **Auto-stimulation of Cdc42 membrane recruitment and activation. (1)** The small G protein Cdc42 is activated (exchanges GDP for GTP) at some basal stochastic rate. **(2)** Activated Cdc42 recruits a complex containing the proteins Bem1, Ste20 and Cdc24. **(3)** Membrane associated Cdc24 (a guanine exchange factor or GEF) activates additional membrane-bound Cdc42. **(4)** As in (2), activated Cdc42 recruits additional Bem1/Ste20/Cdc42 complexes, completing the positive feedback loop (Kozubkowski et al 2008, Johnson et al. 2011).

**4. References**

Anders, K.R., and Botstein, D. (2001). Dominant-lethal alpha-tubulin mutants defective in microtubule depolymerization in yeast. Mol Biol Cell *12*, 3973-3986.

Arkin, A., Ross, J., and McAdams, H.H. (1998). Stochastic kinetic analysis of developmental pathway bifurcation in phage lambda-infected Escherichia coli cells. Genetics *149*, 1633-1648.

Ausubel, F.M., Brent, R., Kingston, R.E., Moore, D.D., Seidman, J.G., Smith, J.A., and Struhl, K. (1987-2017). Current protocols in molecular biology. (New York, N.Y.: John Wiley & Sons, Inc.).

Bishop, A.C., Ubersax, J.A., Petsch, D.T., Matheos, D.P., Gray, N.S., Blethrow, J., Shimizu, E., Tsien, J.Z., Schultz, P.G., Rose, M.D., Wood, J.L., Morgan, D.O., and Shokat, K.M. (2000). A chemical switch for inhibitor-sensitive alleles of any protein kinase. Nature *407*, 395-401.

Choi, P.J., Cai, L., Frieda, K., and Xie, X.S. (2008). A stochastic single-molecule event triggers phenotype switching of a bacterial cell. Science *322*, 442-446.

Cohen, A.A., Geva-Zatorsky, N., Eden, E., Frenkel-Morgenstern, M., Issaeva, I., Sigal, A., Milo, R., Cohen-Saidon, C., Liron, Y., Kam, Z., Cohen, L., Danon, T., Perzov, N., and Alon, U. (2008). Dynamic proteomics of individual cancer cells in response to a drug. Science *322*, 1511-1516.

Colman-Lerner, A., Gordon, A., Serra, E., Chin, T., Resnekov, O., Endy, D., Pesce, C.G., and Brent, R. (2005). Regulated cell-to-cell variation in a cell-fate decision system. Nature *437*, 699-706.

Delbrück, M. (1945). The burst size distribution in the growth of bacterial viruses (bacteriophages). J Bacteriol *50*, 131-135.

Elowitz, M.B., Levine, A.J., Siggia, E.D., and Swain, P.S. (2002). Stochastic gene expression in a single cell. Science *297*, 1183-1186.

Gelin-Licht, R., Paliwal, S., Conlon, P., Levchenko, A., and Gerst, J.E. (2012). Scp160-dependent mRNA trafficking mediates pheromone gradient sensing and chemotropism in yeast. Cell Rep *1*, 483-494.

Giaever, G., and Nislow, C. (2014). The yeast deletion collection: a decade of functional genomics. Genetics *197*, 451-465.

Goldstein, A.L., and McCusker, J.H. (1999). Three new dominant drug resistance cassettes for gene disruption in Saccharomyces cerevisiae. Yeast *15*, 1541-1553.

Goldstein, A.L., Pan, X., and McCusker, J.H. (1999). Heterologous URA3MX cassettes for gene replacement in Saccharomyces cerevisiae. Yeast *15*, 507-511.

Gordon, A., Colman-Lerner, A., Chin, T.E., Benjamin, K.R., Yu, R.C., and Brent, R. (2007). Single-cell quantification of molecules and rates using open-source microscope-based cytometry. Nat Methods *4*, 175-181.

Guthrie, C., and Fink, G.R. (1991). Methods in Enzymology, Guide to Yeast Genetics and Molecular Biology (San Diego, California 92101: Academic Press).

Hittinger, C., and Carroll, S. (2007). Gene duplication and the adaptive evolution of a classic genetic switch. Nature *449*, 677-681.

Hughes, T.R., Roberts, C.J., Dai, H., Jones, A.R., Meyer, M.R., Slade, D., Burchard, J., Dow, S., Ward, T.R., Kidd, M.J., Friend, S.H., and Marton, M.J. (2000). Widespread aneuploidy revealed by DNA microarray expression profiling. Nat Genet *25*, 333-337.

Huh, W.K., Falvo, J.V., Gerke, L.C., Carroll, A.S., Howson, R.W., Weissman, J.S., and O'Shea, E.K. (2003). Global analysis of protein localization in budding yeast. Nature *425*, 686-691.

Johnson, J.M., Jin, M., and Lew, D.J. (2011). Symmetry breaking and the establishment of cell polarity in budding yeast. Curr Opin Genet Dev *21*, 740-746.

Kozubowski, L., Saito, K., Johnson, J.M., Howell, A.S., Zyla, T.R., and Lew, D.J. (2008). Symmetry-breaking polarization driven by a Cdc42p GEF-PAK complex. Curr Biol *18*, 1719-1726.

Longtine, M.S., Fares, H., and Pringle, J.R. (1998a). Role of the yeast Gin4p protein kinase in septin assembly and the relationship between septin assembly and septin function. JCell Biol *143*, 719-736.

Longtine, M.S., McKenzie, A., III, Demarini, D.J., Shah, N.G., Wach, A., Brachat, A., Philippsen, P., and Pringle, J.R. (1998b). Additional modules for versatile and economical PCR-based gene deletion and modification in Saccharomyces cerevisiae. Yeast *14*, 953-961.

Lutz, R., and Bujard, H. (1997). Independent and tight regulation of transcriptional units in Escherichia coli via the LacR/O, the TetR/O and AraC/I1-I2 regulatory elements. Nucleic Acids Res *25*, 1203-1210.

Maddox, P., Chin, E., Mallavarapu, A., Yeh, E., Salmon, E.D., and Bloom, K. (1999). Microtubule dynamics from mating through the first zygotic division in the budding yeast Saccharomyces cerevisiae. J Cell Biol *144*, 977-987.

Maddox, P.S., Stemple, J.K., Satterwhite, L., Salmon, E.D., and Bloom, K. (2003). The minus end-directed motor Kar3 is required for coupling dynamic microtubule plus ends to the cortical shmoo tip in budding yeast. Curr Biol *13*, 1423-1428.

McAdams, H.H., and Arkin, A. (1997). Stochastic mechanisms in gene expression. Proc Natl Acad Sci U S A *94*, 814-819.

Meluh, P.B., and Rose, M.D. (1990). KAR3, a kinesin-related gene required for yeast nuclear fusion. Cell *60*, 1029-1041.

Ohya, Y., Sese, J., Yukawa, M., Sano, F., Nakatani, Y., Saito, T.L., Saka, A., Fukuda, T., Ishihara, S., Oka, S., Suzuki, G., Watanabe, M., Hirata, A., Ohtani, M., Sawai, H., Fraysse, N., Latge, J.P., Francois, J.M., Aebi, M., Tanaka, S.*, et al.* (2005). High-dimensional and large-scale phenotyping of yeast mutants. Proc Natl Acad Sci U S A *102*, 19015-19020.

Sikorski, R.S., and Hieter, P. (1989). A system of shuttle vectors and yeast host strains designed for efficient manipulation of DNA in Saccharomyces cerevisiae. Genetics *122*, 19-27.

Spencer, S.L., Gaudet, S., Albeck, J.G., Burke, J.M., and Sorger, P.K. (2009). Non-genetic origins of cell-to-cell variability in TRAIL-induced apoptosis. Nature *459*, 428-432.

Spudich, J.L., and Koshland, D.E., Jr. (1976). Non-genetic individuality: chance in the single cell. Nature *262*, 467-471.

St-Pierre, F., and Endy, D. (2008). Determination of cell fate selection during phage lambda infection. Proc Natl Acad Sci U S A *105*, 20705-20710.

Takahashi, S., and Pryciak, P.M. (2008). Membrane localization of scaffold proteins promotes graded signaling in the yeast MAP kinase cascade. Curr Biol *18*, 1184-1191.

Tong, A., and Boone, C. (2007). High-Throughput Strain Construction and Systematic Synthetic Lethal Screening in Saccharomyces cerevisiae. In Yeast Gene Analysis (Elsevier Ltd), pp. 369-386 and 706-707

Tong, A.H., Lesage, G., Bader, G.D., Ding, H., Xu, H., Xin, X., Young, J., Berriz, G.F., Brost, R.L., Chang, M., Chen, Y., Cheng, X., Chua, G., Friesen, H., Goldberg, D.S., Haynes, J., Humphries, C., He, G., Hussein, S., Ke, L.*, et al.* (2004). Global mapping of the yeast genetic interaction network. Science *303*, 808-813.

Vallen, E.A., Hiller, M.A., Scherson, T.Y., and Rose, M.D. (1992). Separate domains of KAR1 mediate distinct functions in mitosis and nuclear fusion. JCell Biol *117*, 1277-1287.

Zaichick, S.V., Metodiev, M.V., Nelson, S.A., Durbrovskyi, O., Draper, E., Cooper, J.A., and Stone, D.E. (2009). The mating-specific Galpha interacts with a kinesin-14 and regulates pheromone-induced nuclear migration in budding yeast. Mol Biol Cell *20*, 2820-2830.

Zhang, G., Li, C., Li, Q., Li, B., Larkin, D.M., Lee, C., Storz, J.F., Antunes, A., Greenwold, M.J., Meredith, R.W., Odeen, A., Cui, J., Zhou, Q., Xu, L., Pan, H., Wang, Z., Jin, L., Zhang, P., Hu, H., Yang, W.*, et al.* (2014). Comparative genomics reveals insights into avian genome evolution and adaptation. Science *346*, 1311-1320.
