# Supplementary material for: Single‐cell profiling screen identifies microtubule‐dependent reduction of variability in signaling
Source: Mol Syst Biol. 2018 Apr 4;14(4):e7390. doi: 10.15252/msb.20167390 (PMC5884679; doi:10.15252/msb.20167390)
Supplement: Supplementary file 2 — Expanded View Figures PDF [file MSB-14-e7390-s002.pdf]

Expanded View Figures

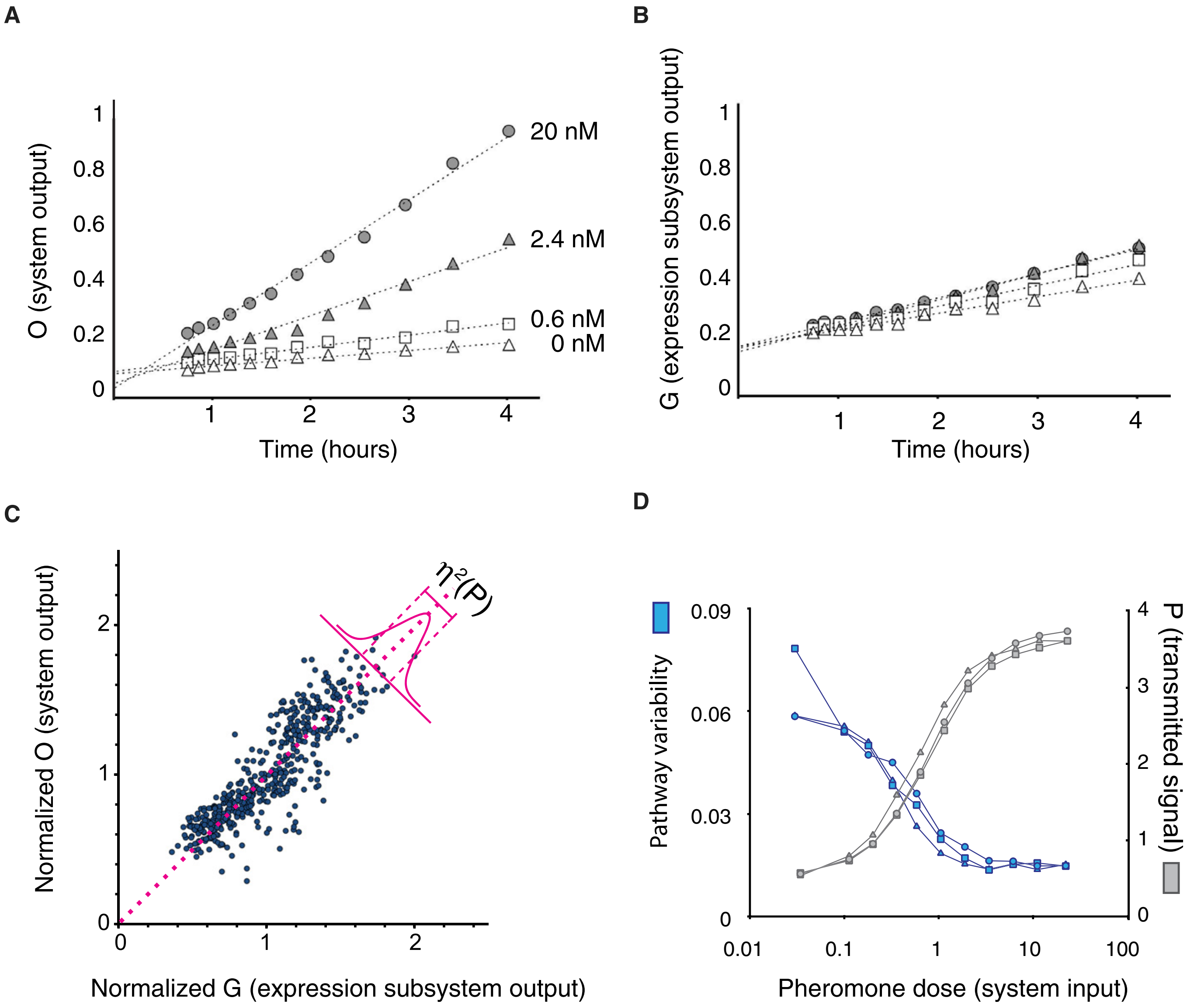

Figure EV1. Time-dependent output and dose response of the reporter genes used in the screen.

We stimulated SGA85 cells with the indicated concentrations of pheromone and measured the accumulated fluorescent protein by flow cytometry as detailed in Materials and Methods.

A, B Average fluorescence output of the pheromone-inducible P_{PRM1}-*mRFP* reporter, **O** (A) and the constitutive P_{ACT1}-*YFP* reporter, **G** (B), in A.U., measured at four different doses over time.

C Estimating pathway variability (η^2(P)). Panel shows a scatter plot, with one point per cell, of P_{PRM1}-*mRFP* vs. P_{ACT1}-*YFP*, in 500 cells stimulated with 20 nM pheromone for 3 h. The amount of pathway variability (η^2(P) + η^2(γ)) (a quantity very close to η^2(P), see Appendix) is a measure of the average width of this distribution of plotted points about the identity line, drawn in pink.

D Dose dependence of pathway variability and output. Plot shows pathway variability η^2(P) + η^2(γ) (blue), and output (gray) in SGA85 cells, as a function of pheromone dose after 180 min.

© 2018 The Authors

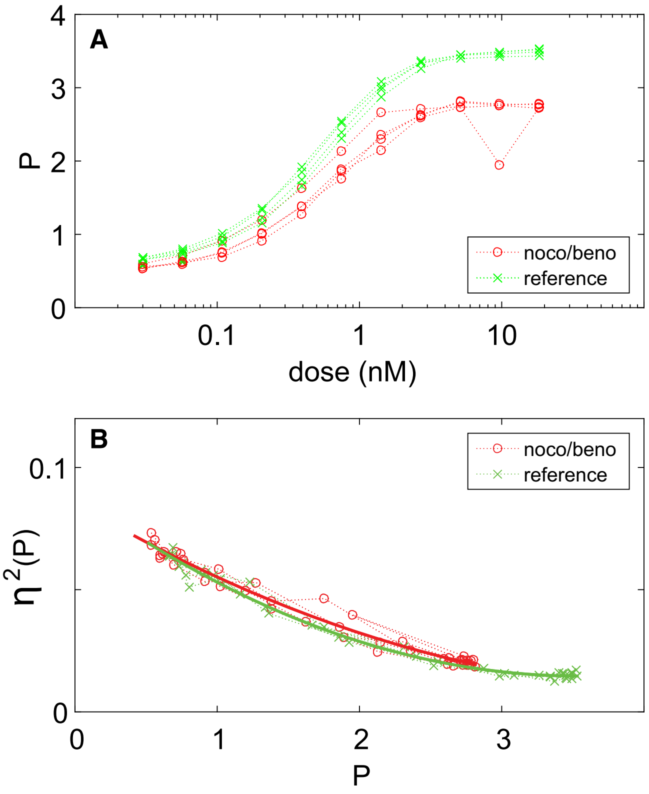

Figure EV2. Chemical inhibitors of microtubule function attenuate signal but do not affect pathway variability.

We treated SGA85 cells with nocodazole and benomyl (Appendix), stimulated PRS activity with the indicated concentrations of pheromone, measured the accumulated fluorescent protein by flow cytometry, and computed $\eta^2(P)$ and P as detailed in Materials and Methods.

A Plot shows P vs. dose.

B Plot shows $\eta^2(P)$ vs. P.

Source data are available online for this figure.

 © 2018 The Authors
