# Supplementary material for: Single‐cell profiling screen identifies microtubule‐dependent reduction of variability in signaling
Source: Mol Syst Biol. 2018 Apr 4;14(4):e7390. doi: 10.15252/msb.20167390 (PMC5884679; doi:10.15252/msb.20167390)
Supplement: Supplementary file 3 — Appendix Table S1 [file MSB-14-e7390-s003.docx]

| **Figures used** | **Description** | **Name** | **Mating type** | **Genotype** | **Reference** |
| --- | --- | --- | --- | --- | --- |
| na | Base strain | BY4741 | *Mata* | S288C, *his3Δ1 leu2Δ0 ura3Δ0 met15Δ0* | Brachmann *et al* (1998) |
| na | Base strain | BY4742 | *Matalpha* | S288C, *his3Δ1 leu2Δ0 ura3Δ0 lys2Δ0* | Brachmann *et al* (1998) |
| na | Base strain | W303-1A | *Mata* | *leu2-3,112 trp1-1 can1-100 ura3-1 ade2-1 his3-11,15* | Thomas and Rothstein (1989) |
| 2 | MATα partner for collection | SGA88 | *Matalpha* | BY4742, *∆can1::P_MFA1_-LEU2 ∆prm1::P_PRM1_-mRFP—natMX4(NAT^R^) cdc28-as2—hphMX4(HygB^R^) ∆bar1:P_PRM1_-CFP—HIS3 ∆lyp1::P_ACT1_-YFP—URA3* | This study |
| 2 | WT, reference for screen | SGA85 | *Mata* | BY4741, *∆can1::P_MFA1_-LEU2 ∆prm1::P_PRM1_-mRFP—natMX4(NAT^R^) cdc28-as2—hphMX4(HygB^R^) ∆bar1:P_PRM1_-CFP—HIS3 ∆lyp1::P_ACT1_-YFP—URA3* | This study |
| 4, 7 | WT, reference for deletions below | GPY4000 | *Mata* | BY4741, *∆bar1 cdc28-as2 ∆prm1::P_PRM1_-mCherry—kanMX6(G418^R^) BMH2::P_BMH2_-YFP—MET15* | This study |
| 4, 7 | *∆bim1* | GPY4001 | *Mata* | GPY4000*, ∆bim1::hphMX4(HygB^R^)* | This study |
| 4, 7 | *∆gim4* | GPY4031 | *Mata* | GPY4000, *∆gim4::natMX4(NAT^R^)* | This study |
| 4 | *∆bim1 ∆gim4* | GPY4036 | *Mata* | GPY4000, *∆bim1::hphMX4(HygB^R^) ∆gim4:: natMX4(NAT^R^)* | This study |
| 7 | *∆fus3* | GPY4004 | *Mata* | GPY4000, *∆fus3::natMX4(NAT^R^)* | This study |
| 7 | *∆fus3 ∆bim1* | GPY4006 | *Mata* | GPY4000, *∆fus3::natMX4(NAT^R^) ∆bim1::hphMX4(HygB^R^)* | This study |
| 7 | *∆fus3 ∆gim4* | GPY4033 | *Mata* | GPY4000, *∆fus3::natMX4(NAT^R^) ∆gim4::hphMX4(HygB^R^)* | This study |
| 7 | *∆kss1* | GPY4038 | *Mata* | GPY4000, *∆kss1:: hphMX4(HygB^R^)* | This study |
| 7 | *∆kss1 ∆bim1* | GPY4053 | *Mata* | GPY4000, *∆kss1::hphMX4(HygB^R^) ∆bim1::URA3MX4* | This study |
| 7 | *∆kss1 ∆gim4* | GPY4051 | *Mata* | GPY4000, *∆kss1::hphMX4(HygB^R^) ∆gim4::natMX4(NAT^R^)* | This study |
| 5 | WT, reference for deletion below | GPY4104 | *Mata* | BY4741, *∆bar1 Cdc28-as2 ∆prm1::P_PRM1_-mCherry—URA3MX6 BMH2::P_BMH2_-YFP—MET15* | This study |
| 5 | *∆cik1* | GPY4123 | *Mata* | GPY4104, *∆cik1:: kanMX6(G418^R^)* | This study |
| 5 | WT, reference for *tub1-828-expessing* | GPY1858 | *Mata* | BY4741, *∆bar1 cdc28-as2 ∆prm1::P_PRM1_-mCherry—kanMX6(G418^R^) BMH2::P_BMH2_-GAL4BD-hER-VP16—LEU2 BMH2::P_BMH2_-YFP—MET15* | This study |
| 5 | *tub1-828* | GPY1873 | *Mata* | GPY1858, *pRB2949 (P_GAL1_-TUB1-828, CEN/ARS, URA3)* | This study |
| na | Base strain used to make strains below | SGA101 | *Mata* | BY4741, *∆can1::P_MFA1_-LEU2 ∆prm1::P_PRM1_-mRFP—natMX4(NAT^R^) cdc28-as2—hphMX4(HygB^R^) ∆bar1:P_PRM1_-CFP—HIS3* | This study |
| 5 | WT, reference for mutants below | SGA103 | *Mata* | SGA101, *BMH2::P_BMH2_-YFP—MET15* | This study |
| 5 | *kar1-∆15* | SGA109 | *Mata* | SGA101, *BMH2::P_BMH2_-YFP—MET15 kar1-∆15* | This study |
| 5 | *kar3-1* | SGA108 | *Mata* | SGA101, *BMH2::P_BMH2_-YFP—MET15 kar3-1* | This study |
| 5 | *∆kar3* | SGA1034 | *Mata* | SGA101, *BMH2::P_BMH2_-YFP—MET15 ∆kar3* | This study |
| 4 | WT, reference for mutants below | GPY123 | *Mata* | SGA101, *HTB2::HTB2-YFP—kanMX6(G418^R^)* | This study |
| 4 | *∆bim1* | GPY4144 | *Mata* | SGA101, *HTB2::HTB2-YFP—kanMX6(G418^R^) ∆bim1::HIS3MX6* | This study |
| 4, | *∆gim4* | GPY4150 | *Mata* | SGA101, *HTB2::HTB2-YFP—kanMX6(G418^R^)* *∆gim4::HIS3MX6* | This study |
| na | Base strain used to construct bypass activators | GPY1858 | *Mata* | BY4741, *∆bar1 cdc28-as2 ∆prm1::P_PRM1_-mCherry— kanMX6(G418^R^) BMH2::P_BMH2_-GAL4BD-hER-VP16—LEU2 BMH2::P_BMH2_-YFP—MET15* | This study |
| 6 | *STE4* Activator | GPY1855 | *Mata* | GPY1858*, HIS3::P_GAL1_—STE4—HIS3* | This study |
| 6 | *STE4* Activator *∆bim1* | GPY1862 | *Mata* | GPY1858, *HIS3::P_GAL1_—STE4—HIS3 ∆bim1::hphMX4(HygB^R^)* | This study |
| 6 | *STE4* Activator *∆gim4* | GPY1898 | *Mata* | GPY1858, *HIS3::P_GAL1_—STE4—HIS3 ∆gim4:: hphMX4(HygB^R^)* | This study |
| 6 | *STE5-CTM* Activator | GPY1915 | *Mata* | GPY1858, *HIS3::P_GAL1_—STE5-CTM—HIS3 ∆ste5:: natMX4(NAT^R^)* | This study |
| 6 | *STE5-CTM* Activator *∆bim1* | GPY1916 | *Mata* | GPY1858, *HIS3::P_GAL1_—STE5-CTM—HIS3 ∆ste5:: natMX4(NAT^R^) ∆bim1::hphMX4(HygB^R^)* | This study |
| 6 | *STE5-CTM* Activator *∆gim4* | GPY1997 | *Mata* | GPY1858, *HIS3::P_GAL1_—STE5-CTM—HIS3 ∆ste5:: natMX4(NAT^R^) ∆gim4:: hphMX4(HygB^R^)* | This study |
| 8 | WT, reference for strains below | MWY003/YPP3662 | *Mata* | W303-1A, *∆bar1 ∆ste5:: natMX4(NAT^R^) trp1-1::STE5-YFP-YFP-YFP--TRP1 (3x)* | Ventura *et al.* (2014) |
| 8 | *∆bim1* | GPY4112 | *Mata* | YPP3662*, ∆bim1::URA3MX4* | This study |
| 8 | *∆gim4* | GPY4113 | *Mata* | YPP3662, *∆gim4::URA3MX4* | This study |
| 8 | WT, reference for *TUB1-828* below | ACL-GP-001 | *Mata* | YPP3662, *BMH2::P_BMH2_-GAL4BD-hER-VP16--LEU2* | This study |
| 8 | *TUB1-828* | GPY4121 | *Mata* | YPP3662, *BMH2::P_BMH2_-GAL4BD-hER-VP16—LEU2 pRB2949 (P_GAL1_-TUB1-828, CEN/ARS, URA3)* | This study |
| App S6 | WT, reference for deletions below | GPY1752 | *Mata* | BY4741, *∆bar1 Cdc28-as2 SPC42-GFP—HIS3MX6 SEC8-mCherry—kanMX6(G418^R^)* | This study |
| App S6 | *∆bim1* | GPY1709 | *Mata* | GPY1752, *∆bim1:: natMX4(NAT^R^)* | This study |
| App S6 | *∆gim4* | GPY1710 | *Mata* | GPY1752, *∆gim4::natMX4(NAT^R^)* | This study |
